# Paving the way to conformationally unravel complex glycopeptide antibiotics by means of Raman optical activity†

**DOI:** 10.1039/d1sc01446c

**Published:** 2021-03-29

**Authors:** Roy Aerts, Jente Vanhove, Wouter Herrebout, Christian Johannessen

**Affiliations:** Department of Chemistry, University of Antwerp Groenenborgerlaan 171 B-2020 Antwerp Belgium christian.johannessen@uantwerpen.be

## Abstract

It is crucial for fundamental physical chemistry techniques to find their application in tackling real-world challenges. Hitherto, Raman optical activity (ROA) spectroscopy is one of the examples where a promising future within the pharmaceutical sector is foreseen, but has not yet been established. Namely, the technique is believed to be able to contribute in investigating the conformational behaviour of drug candidates. We, herein, strive towards the alignment of the ROA analysis outcome and the pharmaceutical expectations by proposing a fresh strategy that ensures a more complete, reliable, and transferable ROA study. The strategy consists of the treatment of the conformational space by means of a principal component analysis (PCA) and a clustering algorithm, succeeded by a thorough ROA spectral analysis and a novel way of estimating the contributions of the different chemical fragments to the total ROA spectral intensities. Here, vancomycin, an antibiotic glycopeptide, has been treated; it is the first antibiotic glycopeptide studied by means of ROA and is a challenging compound in ROA terms. By applying our approach we discover that ROA is capable of independently identifying the correct conformation of vancomycin in aqueous solution. In addition, we have a clear idea of what ROA can and cannot tell us regarding glycopeptides. Finally, the glycopeptide class turns out to be a spectroscopically curious case, as its spectral responses are unlike the typical ROA spectral responses of peptides and carbohydrates. This preludes future ROA studies of this intriguing molecular class.

## Abstract 2

Raman optical activity tackles the complex conformational space of glycopeptide antibiotics.

## Graphical abstract

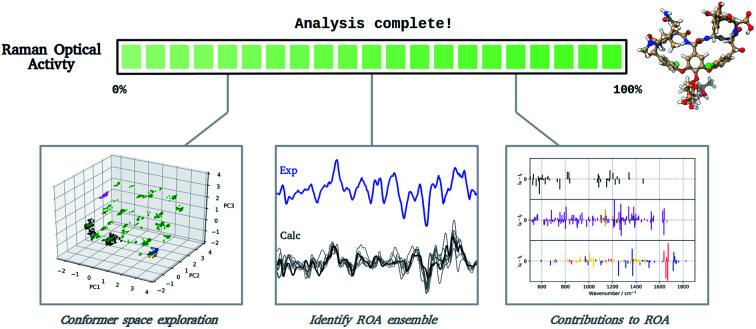

## 1 Introduction

Raman optical activity (ROA) belongs to the set of chiroptical techniques that are typically applied to characterize chiral systems. It makes use of the preferential Raman scattering of right- or left circularly polarized photons by a chiral sample. By measuring the difference in intensity between the two polarization states during the ROA experiment, information regarding the molecular absolute configuration (AC) and the conformation can be obtained.^1–8^ One of the main applications of ROA is the empirical determination of the structural aspects of proteins, such as their secondary structure.^6–8^ For small organic (bio)molecules (peptides, carbohydrates, natural products) the ROA spectra have proven to be substantially more difficult to interpret solely based on empirical data. From the implementation of ROA intensity calculations in density functional theory (DFT) level programs onwards many structural characterization studies of peptides^9–19^ and carbohydrates^20–26^ have been published. Consequently, we now have a clear understanding of the spectral response to the conformational variability in peptides and carbohydrates as well as how to best approach these types of systems. This together with the continuous increase in computing power allows the exploration of applying the ROA technique to study more complex molecular systems by combining experiments and calculations. Specifically, as ROA and the pharmaceutical world have a shared interest in peptide- and carbohydrate-based compounds, ROA is believed to be capable of aiding in the elucidation of the conformational behaviour that is required for rationalizing drug design and mechanisms of action. Therefore, a thorough evaluation thereof is of pivotal importance.

The standard approach that has been established for applying chiroptical techniques in the conformational determination of (relatively) small compound runs as follows: (1) record an experimental spectrum, (2) conduct a conformational analysis by collecting experimentally available ensembles and/or use conformer generator programs (3) optimize the geometries and calculate spectra with DFT methods, and (4) identify the best matching single-conformer or Boltzmann-weighed spectrum with the experiment.^5,27–29^ Adhering to this strategy is a very effective way to answer the central question of what (set of) conformation(s) is adopted by the compound. This approach is, however, not infallible, especially with a growing molecular complexity of the compounds. It might be that the computed spectra of ignored conformations – because they were not sampled in the first place or they vanish during the Boltzmann averaging – compare similarly well with the experiment. In this case ROA cannot distinguish the concerned geometries. This aspect is not generally tested during a standard ROA analysis. Furthermore, other than finding the exact conformation in solution, no direct relationships between geometrical features and ROA bands can be established since the spectral responses towards small and large geometrical changes are not investigated. This prevents a clear insight of what exact information is contained within the ROA spectrum and the transfer of knowledge to related systems. The pharmaceutical sector, however, expects that a structural characterization technique provides reliable answers and requires to know how and where exactly it can aid during the structural characterization of a bioactive compound. In other words, the misalignment between standard ROA study outputs and the demands of the pharmaceutical sector hampers the general application of ROA in pharmacy.

**Fig. 1:**
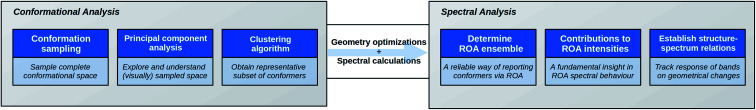
A scheme of the strategy put forward in this contribution for the Raman optical activity (ROA) analysis of complex compounds.

As a response to the above-mentioned concerns, the increasing molecular complexity, and to meet with the application's expectations, we herein expound a novel ROA strategy, summarized in Fig. 1. An as geometrically diverse and complete as possible conformational ensemble of the compound is collected. A principal component analysis (PCA) is applied in order to explore the sampled conformational space. Subsequently, a subset of representative conformations for the complete sampled space is selected by using a *k*-means clustering algorithm. Due to the dimension reduction by the PCA, the visualization of the conformational space becomes possible, providing a unique view on the conformational sampling, the selection of the clustering algorithm, and the general structural effects of DFT-level geometry optimizations. Moreover, this particular treatment of the conformational analysis step opens all geometrical registers for the structure–spectrum analysis at a later stage, and prevents the focus on specifically selected geometrical features based on chemical intuition. During the analysis, a way of establishing a ROA determined conformational ensemble, equivalent to the NMR ensemble, based on the spectral overlap integrals between the computed spectra of all representative structures and the experimental one is presented. Finally, the contributions to the ROA intensity of the different chemical parts of the compound, *e.g.* the peptidic and the carbohydrates part, are determined. Not only does this allow the rationalization of the spectral observations, but it also aids in estimating how and to what extent ROA will be useful in the structural study of the molecular class represented by the compound.

**Fig. 2:**
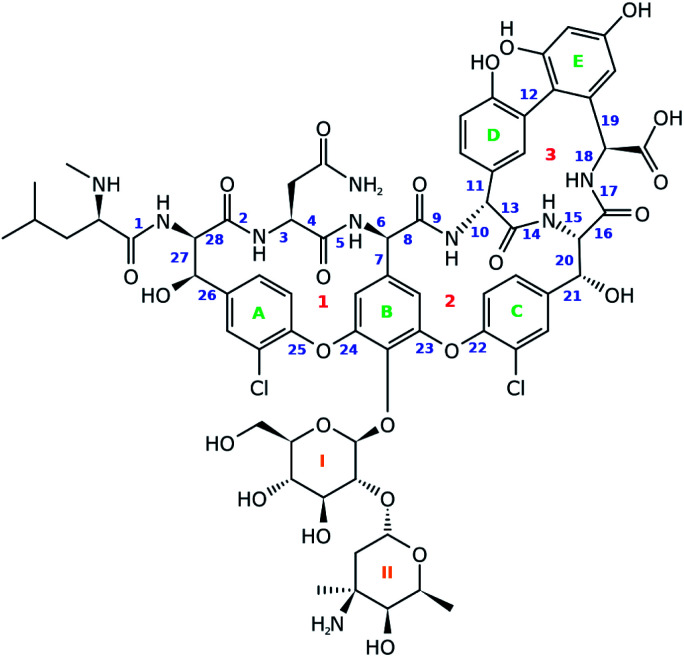
The chemical structure of vancomycin. The blue numbers indicate the bonds around which the dihedral angles are used in the geometrical analysis. The green and orange labels represent the five aromatic rings and two carbohydrate monomers. The red numbers are indications for the three cycles that are present in vancomycin.

For this work vancomycin, an antibiotic of the glycopeptide type, has been carefully selected (see Fig. 2). The molecule is chemically complex in that it hosts seven unnatural amino acid building blocks, five aromatic rings and a disaccharide, consisting of the monomers glucose and vancosamine. Interestingly, vancomycin still receives a lot of attention from the pharmaceutical and medical world, despite its first isolation from *Streptomyces orientalis* dating back to 1956.^30–32^ In fact, alternative antibiotic agents were rapidly favoured over vancomycin because they displayed less severe toxic effects and were more efficacious. However, due to the occurrence of penicillin- and methicillin-resistant *Staphylococcus aureus* bacterial stains, the clinical use of vancomycin resurged.^31,33^ This is reflected in the increasing number of recent studies searching for ways to ameliorate the administration of the antibiotic.^31,34,35^ Also in search for novel related antibiotics, vancomycin is and continues to be an interesting molecular scaffold.^32,36–38^ In addition, the conformation of the molecule has been extensively studied, making it an exquisite system for the evaluation of the performance of the ROA technique.^39–42^ In this study we report for the first time the ROA spectral recordings and analysis of an antibiotic glycopeptide. Coming forth from our analysis, this type of molecule turns out to be spectroscopically particularly interesting, as it breaks with the spectral trends observed for peptides and carbohydrates.

## 2 Methodology

### 2.1 Principal component analysis and clustering algorithm

A principal component analysis (PCA) was applied in order to reduce the complexity of the conformational ensemble (see Section S1.1,† for the details of the conformational sampling).^43^ The method finds the principal components (PCs) that successively maximize the variance through linear combinations of the columns in an (*n*-conformations × *p*-variables) data matrix. PCA mathematically drops into an eigenproblem, where the *p*-eigenvectors represent the coefficients in the linear combination (the PC loadings) and the *p*-eigenvalues indicate the corresponding variance that is stored within the PC. The result of applying the linear combination to the original data matrix are called the PC scores. Prior to the PCA and/or clustering algorithm applied on the conformational ensemble, a representation of the geometry needs to be chosen. Often the 3*N*-Cartesian coordinates are used, but the limitation of this representation is that complete elimination of the overall rotation is not achievable.^44^ A remedy for this is to switch to internal coordinates. Here, we opt to use the dihedral angles and adopt the dihedral angle PCA (dPCA) strategy to overcome the issue of the periodic character of the torsional values.^44,45^ The dPCA method involves the transformation of the space of dihedral angles {*φ*_*n*_} to the metric coordinate space {*x*_*n*_ = cos *φ*_*n*_, *y*_*n*_ = sin *φ*_*n*_}. This is equivalent to using the vector (*x*, *y*) on the unit circle corresponding to dihedral angle *φ*. The characteristics of this mathematical transformation are extensively described by Altis *et al.*^45^ A crucial property of the transformation is that it represents a bijection, which implies that there is a one-to-one correspondence between the *N*-dimensional space and the 2*N*-dimensional vector space. In the case of vancomycin, 28 dihedral angles were taken into account in the input matrix, highlighted by a blue number in Fig. 2. Here, only the tricyclic system in vancomycin, and not the sugar and peptide side chain, is taken into account because this part of the molecule defines the core of the geometry. Also, the variability of the dihedral angles will undesirably obscure the dihedral angles in the tricyclic system. By visually inspecting the 3D PC scores plot of PCA (representing 70% of the variance in the original data), approximately 55 different conformational groups could be identified. Therefore, a *k*-means clustering algorithm was applied to the PC scores of the first three PC's to form 55 conformational clusters.^46,47^ For each cluster, a representative was selected by finding the conformation that lays closest to the centroid of its cluster in the 3 PC scores coordinate system.

### 2.2 Calculating contributions to the ROA and Raman spectra

All the 55 above-mentioned selected conformations were submitted to DFT-level geometry optimizations and spectral calculations using the Gaussian 16 rev.03 program.^48^ The computational details can be found in Section S1.2.† During the analysis of all the calculated spectral data of vancomycin, it is crucial to understand the contributions of each of the three chemical entities – the peptide, aromatic rings, and sugars – throughout the complete ROA spectrum. Typically one inspects the individual normal modes visually, assigning each normal mode to a specific part of the molecule (localized modes) or to several parts of the molecule (delocalized modes). A visual inspection of all the normal modes of all the considered conformations is inefficient. Therefore, an intuitive automation of a visual assignment has been devised, written in Python3.6.8. Its algorithm goes as follows:

(1) The *x*-, *y*- and *z*-components of the displacement vectors during the vibration per atom per normal mode are extracted from the Gaussian output files, and the magnitude of each of the displacement vectors is determined.

(2) For each normal mode, the atoms displaying a displacement vector with a magnitude larger than 0.1 are retained. This threshold is set to prevent that negligible atomic displacements are counted as a genuine vibration. If no threshold is set, too many normal modes will be labelled as a combination of vibrations throughout the whole or different parts of the molecule.

(3) The atom list is compared to atom lists indicating what atom belongs to the peptidic, aromatic, or sugar entity of the molecule. The normal mode is hence assigned to one of the following categories: peptide + aromat + sugar (PAS), peptide + aromat (PA), peptide + sugar (PS), aromat + sugar (AS), peptide (P), aromat (A), sugar (S).

Once the assignment has been performed, for each individual Gaussian output file a normal mode picture can be drawn as depicted in Fig. S1 (ROA) and S2 (Raman).† Here, the normal mode assignment has been applied to the 55 conformation Gaussian output files (see Fig. S3 and S4† for a similar picture as Fig. S1 and S2,† but for all conformations together). Finally, the contribution of each of the six categories to the total ROA and Raman intensity within one of the seven spectral regions (<800 cm^−1^, 800–1100 cm^−1^, 1100–1300 cm^−1^, 1300–1420 cm^−1^, 1420–1600 cm^−1^, 1600–1685 cm^−1^, 1685–1900 cm^−1^; these regions have been selected based on visually analyzing Fig. S1–S4†) can be estimated. This was done by summing all the intensities carried by the normal modes per category and spectral region (based on the normal mode wavenumber). For ROA intensities an additional distinction of positive and negative intensities was made. Then, the total intensity (for ROA positive and negative together) over all categories for each of the seven spectral regions has been determined. These are then used to express the contributions as a percentage of intensity with respect to the total intensity in that spectral region. The results are collected in a grouped bar plot. During this analysis no prior scaling factor has been applied on the wavenumbers of the normal modes.

## 3 Results and discussion

### 3.1 Conformational analysis and principal component analysis visualizations

**Fig. 3:**
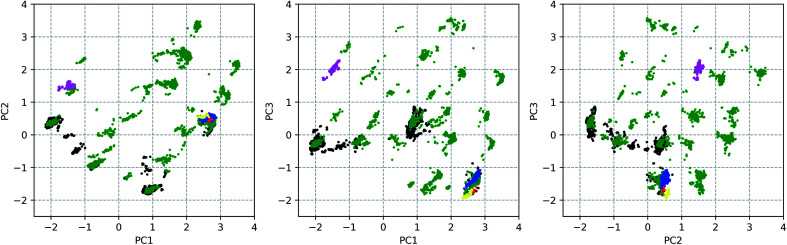
The principal component (PC) scores of all the 12 377 conformations along the three first PCs. The colour indicates the origin of the conformation: XRD (red; 6 conformations), NMR (yellow; 160 conformations), Pcmodel (green; 3452 conformations), CONFLEX (black; 7768 conformation), CREST-1 (blue; 519 conformations), CREST-2 (magenta; 472 conformations).

The sampled conformational ensemble contains 12 377 conformations of vancomycin, collected through existing experimental geometries (6 XRD,^40^ 160 NMR^41^), and computational conformer generators (3452 Pcmodel, 7768 CONFLEX, 519 CREST-1, 472 CREST-2). Pcmodel^49^ and CONFLEX^50^ are two types of conformational search programs that are based on classical force fields and are routinely used to generate an adequate conformational ensemble for chiroptical spectroscopy analyses.^4,5^ On the other hand, CREST evaluates energies on a semi-empirical quantum chemical level of theory and searches conformers by a metadynamics combined with a genetic Z-matrix crossing algorithm.^51^ The notion behind employing a variety of conformer search algorithms (see Section S1.1†) is to find any conformation that is adoptable by vancomycin, regardless of their origin (experimental or through a generator) or their energy, delivering an as structurally extensive ensemble as possible. This helps to grasp the conformational possibilities of vancomycin and opens all registers during the subsequent ROA spectral analysis. A principal component analysis (PCA) was applied on the 12 377 sampled conformations, with the 28 dihedral angles, labelled blue, as input (see Fig. 2). The portion of the total variance in the original data explained by the first two, three and nine principal components (PCs) together is respectively 61, 71, and 90% (see scree plot in Fig. S6†). Typically, for using PCA to reduce the dimension of the data set, the cumulative variance of the first *n* selected PCs should surpass ∼70%.^43^ Therefore, we decided to consider the first three PCs only, restricting the geometrical analysis to three dimensions, which is advantageous because it can be readily visualized. Fig. 3 depicts the PC scores of the 12 377 conformations along the first three PCs. The 3D equivalent can be consulted in Fig. S7.† By visualizing along these three PCs, a representation for the conformational space of vancomycin is constructed. In that space, clear clouds can be observed, which indicate the presence of well-defined conformational groups.

From the 3D PC scores plot visualization (Fig. 3), the evaluation of the structures obtained from the various conformational searches and experimental ensembles can be performed, using the relative positions. Pcmodel samples in approximately all regions of the conformational space where conformations are found and finds many unique conformational groups. Although the CONFLEX generator delivered many more structures than any other conformer generator, it did sample more locally than Pcmodel. All experimental conformations resemble each other and have similar PC scores because all conformations in the experimental ensembles are similar. The low-energy output ensemble generated during the first CREST run (CREST-1; blue dots in Fig. 3) stayed in the proximity of the input XRD geometry. The second CREST (CREST-2) run started with an input geometry situated at the coordinates (0.39, 0.12, 0.96) in the 3D PC coordinate system. Its output ensemble (magenta dots in Fig. 3), however, did not stay in the vicinity of the input and shifted towards a region around the PC coordinates (−1.50, 1.50, 2.00). This shows that CREST searches depend on the input structure. None of the two CREST conformational searches explored new areas in the conformational space of vancomycin. In fact, in a similar way any additional conformational ensembles – originating from *e.g.* alternative conformer generators – can be evaluated (visually) by mapping their geometries in the existing conformational space representation and comparing their positions with the already sampled conformations.

Next, it is important to understand on what basis the separation of the geometries happened along the PCs. The PCs should be regarded as artificial geometrical variables that are created by a linear combination of the original variables (the vector (*x*, *y*) on the unit circle of dihedral angle *φ*). Therefore, the coefficients that were used to construct each of the three PCs, *i.e.* the PC loadings, should be examined. The PC loadings graph and the corresponding biplot are added as Fig. S8 and S9,† respectively. If the absolute value of the PC loading is larger than the value that all PC loadings would have if every original variable were to contribute equally in the linear combination to form the PC, then that variable is considered to contribute significantly to that PC. For instance, PC1 is a combination of the dihedral angles 8 (*x*, *y*), 10 (*x*, *y*), 11 (*y*), 13 (*x*, *y*), 14 (*x*), 16 (*x*, *y*), and 18 (*x*, *y*). Doing this for all three PCs allows us to list the important dihedral angles – that is dihedral angles that vary the most among the different conformations: 4, 7, 8, 10, 11, 13, 14, 16, 18, 21, 22, 23, 25, and 26. That 14 out of 28 dihedral angles define the separation of the geometries between conformational groups demonstrates that the conformational space that has been sampled is complex; variance is present for many dihedral angles among the conformations (Fig. S11 and S12† depict the dihedral angle distributions of the complete conformational ensemble for all the 28 torsions). Noticeable is that five of those torsional angles are defining the geometry of ring system 3 (red indicators in Fig. 2). The dihedral angles 4, 8, and 10 represent changes in geometries throughout parts of the peptidic backbone. Furthermore, the dihedral angles 7, 21, 22, 23, 25, and 26 run over bonds just next to the aromatic systems A, B and C (green in Fig. 2), indicating their variability in the exact (relative) positioning amongst all conformations. The strongest contributor to the three PCs combined is dihedral angle 14, which is surprisingly an *ω*-angle of the peptidic bond. One would expect peptidic bonds to favour the *trans* configuration, but 23% of the conformations are *cis* configurated. All of the experimental conformations have a *cis* peptidic bond. A visualization of the separation in the PC conformational space representation based on the *x*-component of 14, as well as a table presenting the *cis*/*trans* ratios for all peptidic bonds are given in the ESI, Fig. S13 and Table S3.† In contrast with this particular *ω*-angle, *cis* configurations are seldom or not present for the five other peptidic bonds.

### 3.2 Conformer selection and geometry optimizations

**Fig. 4:**
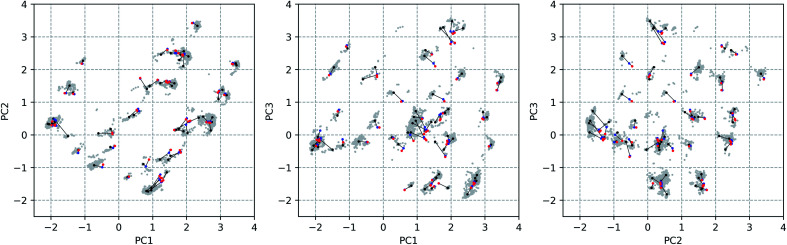
The principal component (PC) scores plot along the three first PCs. The 12 377 conformations are indicated by grey dots. The black dots represent the 55 selected conformations through a *k*-means clustering algorithm. The effect of the first geometry optimization at the B3PW91/6-31G(d,p) level of theory (blue dot connected with the black dot by a black line) on each of the 55 input structures and the successive geometry optimization at B3PW91/6-31++G(d,p) (red dot connected with previous blue dot by a black line) is presented.

Through a visual inspection of Fig. 3 and S7,† approximately 55 conformational groups (clouds in the 3D PC representation) can be identified. As this is quite a large amount of conformations and because no different conformations could be found during other attempts of conformational searches, we assume here to have approximately sampled the complete conformational space that is accessible to vancomycin. A total of 55 representative conformations out of 12 377 were selected by a *k*-means clustering algorithm in order to reduce the subsequent computational problem. Their positions in the conformational space are shown in Fig. 4 as black dots (Fig. S14 and S15† depict the black dots only for clarity). Visually, it becomes evident that the clustering algorithm performed as desired: it samples uniformly throughout the complete sampled conformational space.

**Fig. 5:**
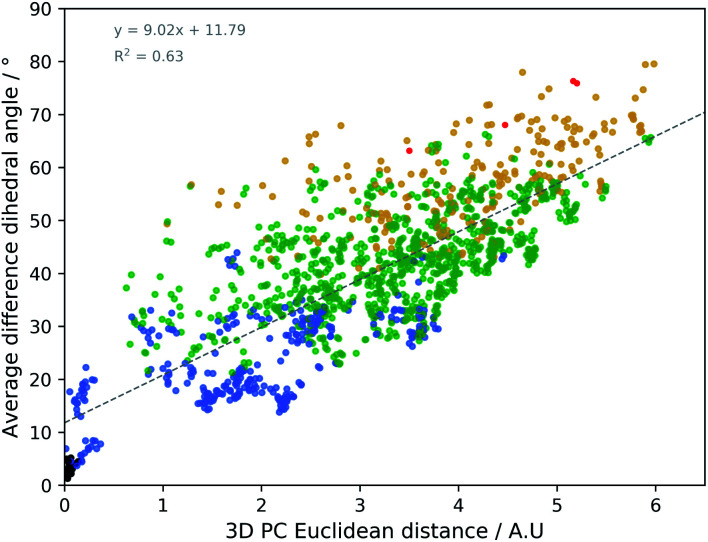
The 3D PC Euclidean distance (in arbitrary units) *versus* the average difference per dihedral angle between two conformations. The comparison is done for all 55 representative, doubly optimized, conformations. The colours indicate that 0 (black; 20 points), 1–7 (blue; 314 points), 8–14 (green; 904 points), 15–21 (yellow; 243 points) or 22–28 (red; 4 points) significantly different (>20°) dihedral angles (out of the 28 that were considered) are to be found between the two compared geometries. The details of the linear regression fit are depicted in grey at the top left of the plot.

The geometrical effect of the two successive geometry optimizations (GO), at the respective B3PW91/6-31G(d,p) and B3PW91/6-31++G(d,p) levels of theory, are visualized in Fig. 4 as blue and red dots. Before the discussion of the effect of the GOs on the PC scores of the representatives, a general insight in the meaning of the 3D PC Euclidean distance should be given. The corresponding average difference in the value of each dihedral angle used in the PCA between each of the 55 representative conformations (after two GOs) was calculated (this implies the comparison between 55^2^ − 55/2 = 1485 conformations), and are traced *versus* the 3D PC Euclidean distance (see Fig. 5). Additionally, for each comparison the number of dihedral angles out of 28 that differed significantly (here a threshold of 20° is used) is determined; in Fig. 5 the dots are given a colour corresponding to how many different torsions were counted (see caption Fig. 5 for meaning of each colour). In general, there is a connection between the 3D PC distance and the conformational difference, illustrated by a reasonable linear regression fit. Since the 3D PC distance is intrinsically different for each comparison, a deviation from (*R*^2^ = 0.63) the linear regression fit is expected. Also, based on the colours, if the two conformations differ in many dihedral angles, it tends to be reflected in the 3D PC distance. It should be noted that exceptions can be found, and this depends on the exact absolute value of the torsional differences and the spread of the conformational difference over a large or small number of dihedral angles. As such, the 3D PC distance can be seen as a measure to express conformational differences in a general way. By inspecting the ordinate of Fig. 5, one can clearly get an impression of the conformational heterogeneity of the complete sampled ensemble: the difference in average dihedral angle between the conformations is mostly between 30–60° and can even go up to 80°.

The effect of DFT-level geometry optimizations on the structure of the 55 representatives can also be evaluated in light of the PC conformational space representation. Upon optimization of each of the representatives, some representatives will converge geometrically, and others will diverge. In general, as apparent from their averages and ranges, the *inter* representative 3D PC distances decrease slightly for the optimized structures (see Table 1), but remain in the same order of magnitude as those of the starting geometries. The occupied conformational space remains similar. However, some representatives are no longer distinguishable after a certain geometry optimization step. If the *inter* representative distance between two conformations drops below half of the lowest *inter* representative distance in the starting conformational space, *i.e.* 0.05 (0.10 divided by two), than the two conformations are no longer distinguishable. By using this threshold, the number of distinguishable conformational groups decreases from 55 to 48 and 47 after the two successive geometry optimizations, respectively. When zooming in on the geometry optimizations for each of the 55 representatives, the average distance travelled in the conformational space due to the first and second GO are 0.31 and 0.07. The order of magnitudes of the geometrical step is smaller than the *inter* representative averages, which quantitatively supports the visual picture: the conformations stay close to their starting geometries relative to the complete conformational space. Additionally, with the aid of Fig. 5 an estimation of the average dihedral angle change throughout the complete conformation as a consequence of each GO can be made. Namely, as the range of PC distances for the first and second GO are 0.04–0.74 and 0.01–0.31, respectively, the estimated average dihedral changes are in the order of 11–18° (first GO) and 11–16° (second GO). The core structure of vancomycin can take on various geometries, but is not easily interconverted. The first GO step is larger (larger average PC distances) because the force field changes drastically from (semi-)classical to DFT-level ones and because an implicit water solvent is applied. The addition of diffuse functions in the basis set in the second GO step in general only induces minor corrections to the geometries. Those minor corrections either continue the geometrical change of the first GO (an angle around 180° between the two vectors that represent the geometrical change in the 3D PC space; see Table S4†), change the direction (angle close to 90°) or partially revert the geometrical change (angle close to 0°). Even after the two GOs, it can be concluded from the 3D PC plot that the potential energy surface remains very complex, hosting many local minima (all the optimized structures had no imaginary frequencies).

**Table 1:** Numerical overview of the effect of the two successive geometry optimizations (GO) on the starting geometries of the 55 representative conformations. In the table, the distance in the 3D PC scores coordinate system is abbreviated as ‘*d*()’ and all have arbitrary units. With the labels ‘to start’ and ‘to opt. 1/2’ are short for with respect to the starting representative geometry and the geometry after a first/second GO, respectively

	Start	Opt. 1	Opt. 2
DFT basis set	—	6-31G(d,p)	6-31++G(d,p)
Average *d*(*inter* repres.)	3.26	3.17	3.16
Range *d*(*inter* repres.)	0.10–6.25	0.02–5.97	0.01–5.98
Conformational groups	55	48	47
Average *d*(to start)	—	0.31	0.32
Range *d*(to start)	—	0.04–0.74	0.06–0.83
Average *d*(to opt. 1)	0.31	—	0.07
Range *d*(to opt. 1)	0.04–0.74	—	0.01–0.31
Average *d*(to opt. 2)	0.32	0.07	—
Range *d*(to opt. 2)	0.06–0.83	0.01–0.31	—

### 3.3 Spectral analysis by using the existing standard approach

**Fig. 6:**
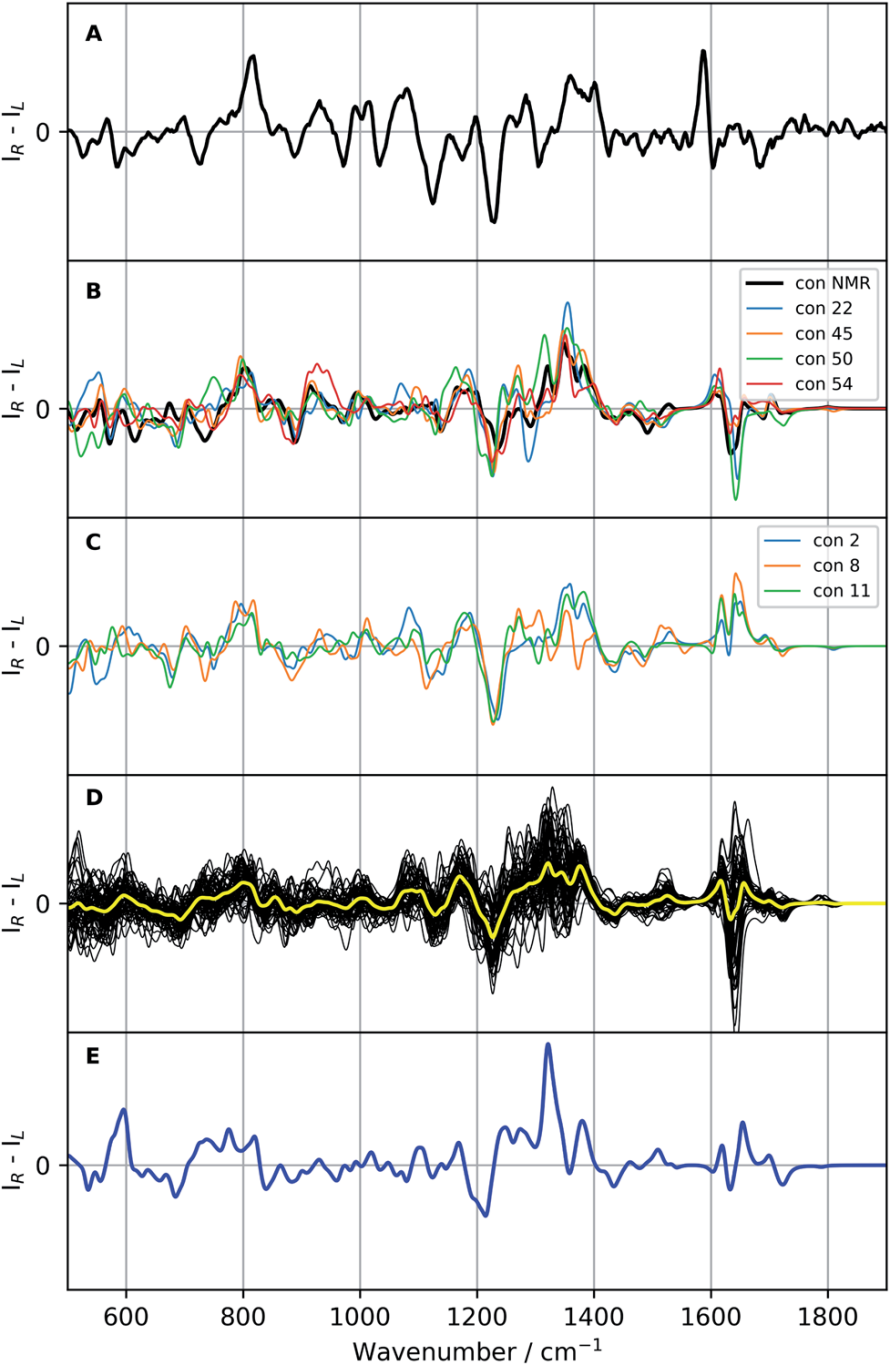
Raman optical activity (ROA) spectra of vancomycin: the experimental ROA spectrum of vancomycin in aqueous solution (A), a selection of calculated spectra (B and C; colour specified in the legend), all calculated ROA spectra (B3PW91/6-31++G(d,p)) of the 55 representatives (black, D) with their average in yellow, and the Boltzmann weighted spectrum (blue; E; the relative enthalpies and weights are specified in Table S5†). A scaling factor of 0.987 was applied on all calculated spectra.

The experimental ROA spectrum of vancomycin in aqueous solution can be found in panel A of Fig. 6 (the Raman spectrum can be found in Fig. S16†). The experimental details of the measurements are detailed in Section S1.3.† What vibrational modes we expect to find from the chemical parts (peptides, aromatic systems and carbohydrates) of vancomycin in the experimental spectrum has been outlined in the literature.^52–54^ For the peptidic part of vancomycin, the important spectral regions are: the amide I region (C=O stretch vibration 1600–1690 cm^−1^), the amide II and III regions (coupled C–N stretch an N–H bending vibrations; II: 1480–1580 cm^−1^; III: 1230–1300 cm^−1^), the backbone skeletal stretch region (C_α_–C, C_α_–C_β_, C_α_–N; 870–1150 cm^−1^) and below 800 cm^−1^ (delocalized normal modes, O–C–N bending, out-of-plane N–H bending, out-of-plane C=O bending).^52^ The vibrational modes of the sugars are visible under 1500 cm^−1^. Between ∼1200–1500 cm^−1^ CH_2_ and C–O–H deformations are found, in the region 950–1200 cm^−1^ C–O and C–C stretches and C–O–H deformations govern, and below 950 cm^−1^ the anomeric region is found.^53^ Finally, the aromatic ring stretches can be found in the regions 1580–1615 cm^−1^ and 1450–1510 cm^−1^.^54^ Additionally, C–H in-plane bends and out-of-plane bends are seen in the regions 950–1225 cm^−1^ and 670–900 cm^−1^, respectively. Some ring distortions will also contribute below 1400 cm^−1^. As expected, the ROA spectrum of vancomycin in its totality contains many well-pronounced bands in the above-mentioned regions. Unfortunately, the ROA pattern does not resemble those of peptides and sugars, rendering it impossible to transfer their known structure–spectrum rules to the vancomycin case.

If the standard approach is used to interpret the experimental data, we are to either compare with the spectrum yielded by a previously experimentally-defined geometry or with a Boltzmann weighted ROA spectrum. In the former case, as only one real conformation was present in the experimental ensemble, especially with respect to the geometrical variability in the complete conformational space (see previous 3D PC conformational space representation), the experiment can be compared to the ROA spectrum of conformation 1, a conformation determined by NMR (see panel B Fig. 6). In the spectral region 500–1400 cm^−1^ the two spectra follow the same ROA pattern. The computed aromatic (∼1550–1600 cm^−1^) and amide I region (∼1620–1750 cm^−1^; carbonyl stretch mode) on the other hand require a larger redshift than the standard 0.987, as becomes evident from the corresponding Raman spectra (see Fig. S16†). For the aromatic region, the optimal scaling factor was determined to be 0.965 by maximizing the overlap integral between the experimental and all calculated Raman spectra in the aromatic region (see Table S6 and Fig. S17†). A similar +/− couplet is present in the aromatic region in both the experiment and the calculation, whereas the amide I region does not match. The spectral comparison using the standard approach is in general satisfying. This is supported by the relatively high overlap integral of 0.48 between the ROA spectrum of conformation 1 and in the experiment (see Table 2; see Section S2† for what overlap integral values are represent a good spectral match in chiroptical spectroscopy). This analysis confirms that the NMR-found conformation is also present in the ROA measured sample. We should point out, however, that the concerned NMR conformation is a slow time-average of many unresolved conformers, whereas the ROA signal arises from a linear superposition of individual resolved conformations. When the approach of the comparison of the Boltzmann weighted spectrum (using the 55 representative conformations) is followed – for instance when no *prior* experimental conformations are available – a very different result is obtained (blue spectrum panel E of Fig. 6). The Boltzmann weights of the three most energetically favourable conformations based on the relative enthalpies (see Table S5†) are 92.0%, 7.0% and 0.8% for respectively conformation 31, 50, and 38. This spectrum has a poor comparison with the experiment, with an overlap integral of only 0.10.

**Table 2:** Overlap integrals between calculated ROA of the specified conformations and the experimental (Exp) and first conformation (NMR; conformation behind NMR structure) in the spectral regions 500–1420 cm^−1^ (reg1), and 1550–1650 cm^−1^ (reg2; scaling factor 0.965). In the last column the 3D distance within the previously discussed PC coordinate system is given. This is an extract of Table S7

Conformation	Exp	Exp (reg1)	Exp (reg2)	NMR	NMR (reg1)	NMR (reg2)	PCA dist. (3D)
1	0.48	0.55	0.03	1.00	1.00	1.00	0.00
2	0.52	0.61	−0.07	0.57	0.66	−0.28	2.32
8	0.46	0.58	0.11	0.35	0.50	−0.46	3.74
11	0.53	0.65	0.24	0.51	0.65	−0.46	1.60
22	0.39	0.46	0.49	0.66	0.67	0.56	0.17
31	0.07	0.09	−0.03	0.20	0.21	0.18	4.46
45	0.51	0.57	0.19	0.76	0.77	0.82	0.13
50	0.35	0.43	0.36	0.62	0.61	0.82	2.82
54	0.47	0.55	0.36	0.30	0.30	0.38	0.17

**Fig. 7:**
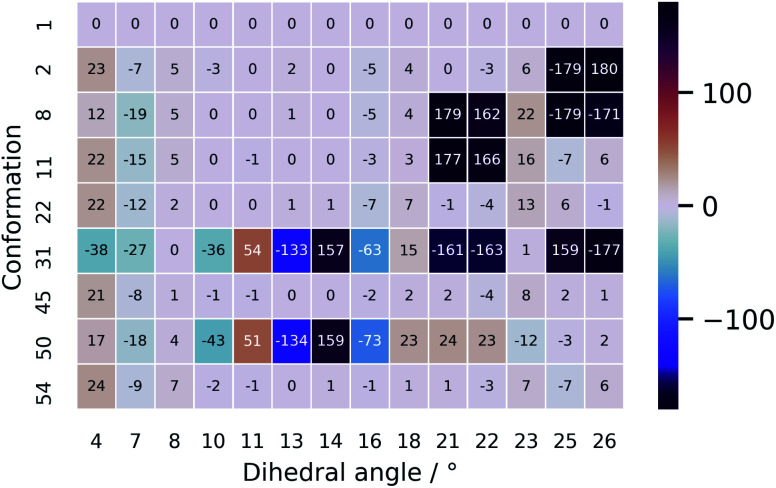
A heatmap representation of the geometries behind the conformations shown in Table 2. Only the dihedral angles that were determined to be important in the PCA are given here. The full geometrical aspects of all 55 conformations can be found in Section S9.†

The unsatisfying result can be explained by examining the geometrical differences between conformations 1 and 31 (only this geometry is taken into account as it has a Boltzmann weight of 92.0%). To facilitate the geometrical discussion, a heatmap representation of the geometries is given in Fig. 7. The two conformations differ in approximately 11 out of the 14 important dihedral angles (see section on PCA). This inevitably evokes a different ROA spectrum when changing the conformation from 1 to 31. What is remarkable is that the experimentally determined conformation 1 does not have a high Boltzmann weight. Here, we believe that this is so because of the *cis*-configured *ω*-angle in one of the peptidic bonds (dihedral angle 14). As this configuration is generally conceived as energetically disfavoured, a conformation, such as conformation 31, with all *trans*-configured peptidic bonds will end up higher in the Boltzmann weight ranking. Here, the spectral disagreement when using their Boltzmann weights is not attributed to the energy uncertainty as discussed by Koenis *et al.*^55^ The reason why vancomycin does adopt the *cis*-configured peptidic bond is because the bond is favourable during the formation of dimers in aqueous solution in the experimental sample.^39^ This emphasizes how a conformational analysis merely based on the Boltzmann weight of monomer conformers can incorrectly grasps the experimental reality, ending up in an inconclusive result.

### 3.4 Extended spectral analysis: an ROA determined conformational ensemble

Since a satisfying match between the experiment and calculation using the experimental conformer is found, in principle the spectral analysis can be concluded here. However, by stopping at this stage, one assumes that there will be no other geometry that yields an ROA spectrum that also matches well with the experiment. If another very different geometry matches equally well, ROA is not capable of distinguishing between those particular conformations. Moreover, at the moment nothing is known with respect to the spectral effect caused my only minor geometrical changes. In other words, we have no idea about the geometrical range that would yield a spectrum matching equally well to the experiment, and therefore, do not know the resolution of our geometrical output. As 55 different geometries are at our disposal from the conformational analysis and selection, we proceed with a more thorough elaboration of the above-mentioned concern. The 55 ROA spectra are depicted in panel D of Fig. 6. As expected, the spectra are very variable, reflecting a significant response of the spectrum on the geometrical changes. Especially the aromatic region (1550–1650 cm^−1^) varies drastically from greatly negative to positive intensities. However, looking at the general trend of the 55 ROA spectra by taking the regular average (yellow curve in panel D of Fig. 6) shows that some spectral regions tend to follow the experiment. For instance, a positive average around 800 cm^−1^, the +/−/+ pattern between 1100–1200 cm^−1^, and the global pattern between 1200 and 1400 cm^−1^ compare well with the experimental ROA spectrum. That the average still follows the experimental spectrum indicates that the general spectral response throughout all conformations is similar to some extent.

All calculated ROA and Raman overlap integrals between the experiment and the 55 calculated ones can be found in Table S7.† The values that will be discussed in the main text are given in Table 2. Only the ROA spectra and their overlaps will be discussed in detail, as the Raman spectra and corresponding overlap integrals are remarkably similar for all the 55 conformations. Looking at the ranges only, it is evident that ROA is much more sensitive to conformational changes than the Raman counterpart. The first important conformation to consider is 11, which has the highest overlap integral (0.53) with the experimental ROA spectrum. As one should be careful not to overvalue the exact overlap integral value, we safely assume that all conformations that have an overlap integral within 0.10 from the maximum of 0.53 should also be considered as satisfying agreements with the experimental spectrum (see Section S2†). This delivers five more conformations: 1 (0.48; discussed previously), 2 (0.52), 8 (0.46), 45 (0.51), and 54 (0.47). From the regional overlap integrals in Table 2 it can be concluded that the high overlap integrals almost always originate from a good spectral comparison in the 500–1400 cm^−1^ region, again emphasizing the high variability in the aromatic region (1550–1650 cm^−1^) throughout the set of calculated ROA spectra. As in this specific case we already know that conformation 1 is the correct one, two more conformations that have a highly similar ROA spectrum to the one of conformation 1 merit to be included in the spectral–geometrical analysis: conformation 22 (0.67) and conformation 50 (0.61).

**Fig. 8:**
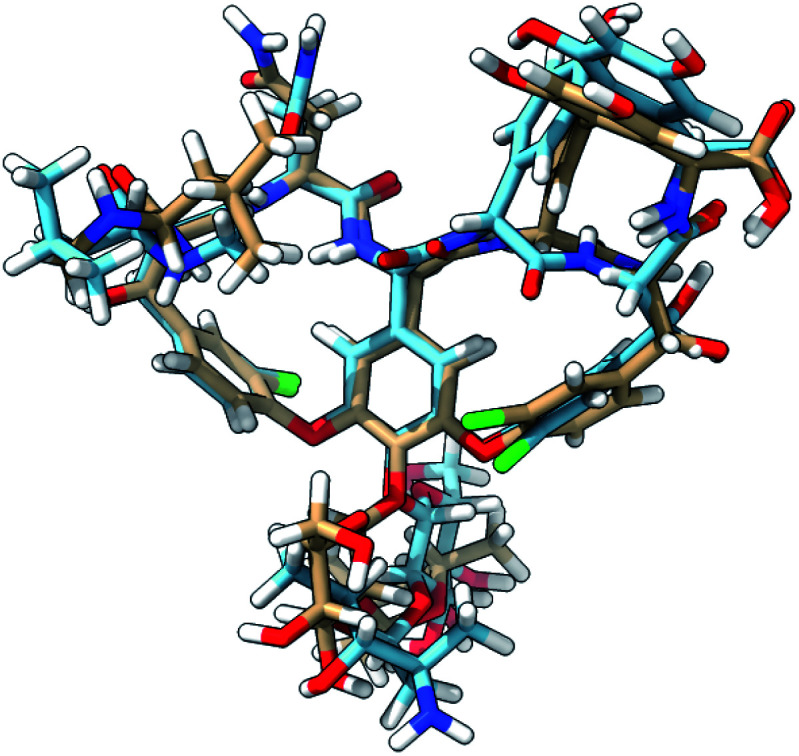
The 3D structure of vancomycin adopting conformation 1 (NMR structure; light brown C atoms) and conformation 50 (light blue C atoms). All of the descriptors of location (front, back, down, up) in the main text are based on the global positioning and orientation of vancomycin as depicted here. The structures were visualized through the UCSF ChimeraX program.^56^

The ROA spectra of all above-mentioned conformations have been depicted in Fig. 6, where all spectra that are alike conformer 1 (*S*_fg_ > 0.60 with respect to conformer 1) are plotted in panels B and C. For the geometrical analysis a dihedral angle heatmap with conformation 1 as reference geometry is depicted in Fig. 7. Conformations 22, 45 and 54 are both geometrically and spectroscopically *quasi* identical to conformation 1. The positive contribution at ∼950 cm^−1^ in the spectrum of conformation 54 is a region where it overlaps better with the experiment than conformation 1. Also, for conformations 45 and 54 it seems that the balance in the +/− couplet around 1620 cm^−1^ mimics better the experimental couplet in the aromatic region. Conformation 22 on the other hand compares spectroscopically very well with conformation 1 (total overlap integral of 0.66), whereas it does not agree convincingly with the experiment due to its relatively lower overlap integral of 0.39. Visually, the lower overlap integral can be explained by switches in the signs of intensity in the spectral areas ∼550 cm^−1^ and ∼750 cm^−1^, and the relatively strong contributions at ∼1300 cm^−1^. Remarkably, the conformations 2, 8 and 11 are geometrically identical to conformation 1, apart from the orientation of the aromatic rings A and C (see Fig. 2). For conformation 1 the chlorine substituent of ring A and C point at the back and the front, respectively (see Fig. 8 to see what spatial perspective is used to look at the structure). For conformation 2 and 11 only one of the two rings flip 180° and for conformation 8 both flip 180°. It appears that these ring flips are translated into a solely positive intensity in the aromatic region of the ROA spectrum, specifically with a +/0/+ pattern. At the same time, the three spectra do overlap well with the experiment, with overlap integrals of 0.52, 0.46, and 0.53. Only for conformation 8, when aromatic rings A and C flip simultaneously, a strong spectral effect is observed between 1250 and 1400 cm^−1^.

The spectrum of the last conformation, 50, matches surprisingly well (*S*_fg_ = 0.62) with conformation 1, and matches reasonably with the experiment (*S*_fg_ = 0.35) for a structure that is quite different from the one of conformation 1 (see Fig. 8). The most radical geometrical difference is the *trans*-configured *ω*-dihedral angle (dihedral angle 14). This change goes hand in hand with different dihedral angles around this peptidic bond, *i.e.* dihedral angles 10, 11, 13, and 16. Furthermore, a minor rotation of the aromatic ring C can be observed, pointing the chlorine more downwards.

Now, ROA can be used as a stand-alone technique to propose an ensemble. In a crude way by looking merely at the *S*_fg_ values with the experimental ROA, that would be the collection of geometries 1, 2, 8, 11, 45 and 54. In this way the ROA experimental data is used to select out of a structurally too broad initial conformational ensemble the set of real geometries present in the sample, analogous in spirit with the generation of NMR ensembles, *e.g.* during the NAMFIS (NMR Analysis of Molecular Flexibility In Solution) protocol.^57–60^ With the aid of ROA an ensemble close to the NMR structure (conformation 1) is captured, but no discrimination between the chlorine atoms on ring A an C pointing either to the front or back can be made. As the true conformation was already known in our case, it should be noted that even though conformation 50 is not captured using *S*_fg_ values with respect to the experiment and is not included in the ensemble, ROA can be insensitive to specific conformational changes, and displays insensitivity in this particular case with respect to the geometrical change represented by conformation 50. Before being able to formulate more specific structure–spectrum relationships in order to unlock the transferability of our conclusions to related products, a deeper understanding of the spectral contributions and normal modes that make up the ROA spectrum is required. Section 3.4 is devoted to this.

It should be briefly noted that the analysis has been based on one-to-one spectral matches. For vancomycin this does not pose any issues as we know that only one major conformations is present in solution. However, if two or several dominant conformations in solution, a mere one-to-one comparison is inadequate. In that case, the analyst can plug in the approach as presented by Bogaerts *et al.*^5^ or by determining the weights of the individual conformers by spectral deconvolution^61,62^ and other fitting algorithms^55^ in our strategy. As the authors cautioned in their respective works, we reiterate that applying either of these methods should be done with a critical mind.

In hindsight, our specific PCA and clustering algorithm approach is an adequate preparation for a subsequent ROA analysis. Namely, the ensemble is sufficiently geometrically diverse to observe clear spectral responses, but is narrow enough to observe the insensitivity of ROA with respect to certain geometrical changes or nuances. If, however, no satisfactory overlap was obtained between the calculated ROA spectra and the experiment, it might be that conformations are missing, and in that case one should return to the first step of the workflow. It should also be noted that this type of analysis has been done from the perspective of the experimental conformation (the NMR structure; conformation 1), and that the same can be done with all of the other representatives as reference. The latter might be particularly interesting for molecular systems for which less is known about the real conformation. Furthermore, we would like to draw the attention to the 3D PCA distances (see last column Table 2 for the conformations discussed in this section). Previously, it was mentioned that these distances can be regarded as an index of geometrical change. The utility of this index, however, depends on the application. For visualizing and characterizing conformational groups and the effect of geometry optimizations, the distances are very useful. In the case of the ROA spectra, no direct relationship between the distance index and the overlap integral of their underlying ROA spectra can be established. That is because the set of underlying geometrical changes differs for each distance. Consequently, the PCA distances cannot serve as a guide for explaining particular geometrical changes or to define structure–spectrum relationships.

### 3.5 Contributions of the peptide, aromatic and sugar entities to the Raman optical activity spectrum

**Fig. 9:**
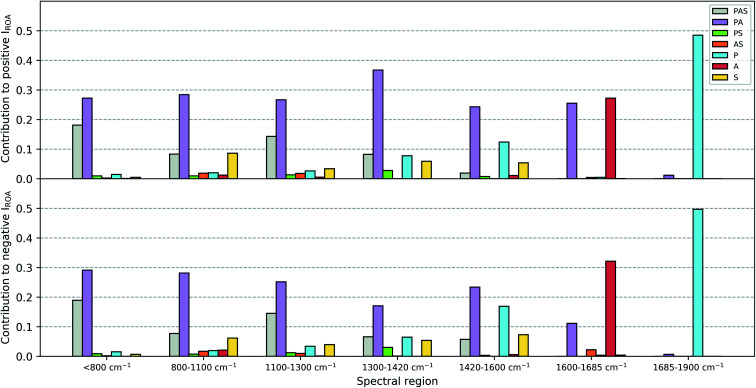
A bar graph to visualize the contributions of the normal modes – assigned to the peptide (P), sugar (S) and aromatic (A) entities, or any combination thereof (PAS, PA, PS, AS) – to the total Raman optical activity intensity in the specified spectral regions. The contribution is split up for positive (top) and negative (bottom) intensities, but the percentage is always calculated with respect to the total (positive plus negative) ROA intensity in that spectral region. The wavenumbers have not been scaled during this analysis.

The contributions of the three major chemical parts of vancomycin (peptide, sugar, aromatic ring) have been estimated as specified in Section 2.2. The spirit behind this calculation is to mimic what one would do during a visual inspection: first identify whether the normal mode vibration is localized or not, and then assign it to its molecular origin. Fig. 9 summarizes the ROA intensity that is carried by the normal modes that have been identified as either peptide, sugar or aromatic ring vibrations, or any combination of the three, and that for all 55 conformations. This is done for several spectral regions, and the negative and positive intensities have been split up (top and bottom of Fig. 9). The Raman counterpart can be consulted in Fig. S5.† A first aspect that can be discussed using this image is the localization of normal modes. Here, we speak of a localized mode when it only involves vibrations arising from one of the three major chemical groups. As such, the five spectral regions below 1600 cm^−1^ are governed by normal modes that have vibrations spread out over the entire or a major part of the molecule. As expected, the normal modes in the spectral regions 1600–1685 cm^−1^ and 1685–1900 cm^−1^, respectively the aromatic and amide I region, are (mainly) localized. A second aspect that is enabled by this figure is the analysis of the contribution of each of the three major chemical parts of vancomycin to the total intensity in the ROA spectrum. From the contributions of all the categories, it becomes immediately evident that the categories involving the aromatic rings (grey, purple, orange, and red bars) carry the largest portions in the total ROA intensity. In contrast, all normal modes with vibrations in the sugar entity (green, orange, and yellow bars) contribute to a much lesser extent, apart from those where the peptide and the aromats are also involved (grey bars). The larger contributions can on one hand be attributed to the fact that there are simply more normal modes in the specific spectral region (and with a specific sign) that have been assigned to the aromatic systems (Table S1† contains the average number of normal modes that have been assigned to each of the six categories). This is, however, not the only reason why the sugar entity contributes less. In Table S2† an estimation of the average contribution of a single normal mode of that category is provided, expressed as a percentage with respect to the total ROA intensity in the specific spectral region. The percentage of the single normal mode contribution for the categories involving sugars is relatively low – except for the aromatic system plus sugar category – compared to the percentages of other categories, and that in all spectral regions. In other words, intrinsically the sugar-assigned normal modes carry a lower ROA intensity.

As the sugar appears to contribute only weakly, any attempt for a structure–spectrum analysis of the carbohydrate specifically risks being inaccurate. Instead, we have calculated the ROA spectra for the 55 vancomycin conformations without their sugar entities. The spectral effect of removing the sugars can be seen in Fig. S19.† Visually, both the single-conformation and the average ROA and Raman spectrum are *quasi* identical. Indeed, this observation is reflected in the high overlap integrals (order of magnitude of ∼0.8–0.9) between each conformation of vancomycin and its counterpart without the sugar (in Table S8†). This result is in stark contrast with other studies, where carbohydrates typically contribute significantly to or dominate the ROA intensities of *e.g.* glycoproteins.^63–67^ It is quite striking to see that the intrinsic contribution of the carbohydrate entities is low, and that it has less to do with the ROA intensity cancellation effect due to the flexibility of the concerned molecular part.^18^

The apparent invisibility of the sugar moiety has important consequences for the utility of ROA as a technique in drug design, where medicinal chemists are interested in the conformation of the sugar and the effect of derivatization (*i.e.* functionalizing the carbohydrate and adding or removing the carbohydrates). Where typically the ROA technique has the advantage of displaying strong and informative carbohydrate signals, for this particular system that is not the case. Consequently, the usual conformational information cannot be extracted. The difference between vancomycin and other systems containing aromatic systems that have been studied by ROA is the presence of a relative large amount of aromatic rings (five) that are locked in certain orientations.^18,28^ Our estimation is that this particular geometrical feature places the aromatic rings in a dominant position in the ROA contributions. If one is, however, not particularly interested in the carbohydrate's conformation, the ROA technique has the benefit of focusing specifically on the rest of the molecule. This brings about two additional advantages. Firstly, in order to correctly predict the conformation of carbohydrates, typically the solvent should explicitly be taken into account during the spectral calculations, typically using a QM/MM spectral calculation.^20,21,23,24,26^ Here, such additional considerations during the computation of the ROA spectra are not necessary. Secondly, it permits us to ignore the conformation of the sugar entities in both the PCA and spectral analysis. This validates our approach and analysis in the previous sections regarding these topics and explains the satisfying theoretical match with the experiment that was obtained without having paid special attention to the carbohydrates.

### 3.6 Structure-Raman optical activity spectrum relationships

Ultimately, the question remains whether with the aid of the current spectral data set more general structure–spectrum relationships can be identified. The majority of normal modes throughout the ROA spectrum are delocalized which allows any geometrical change to induce shifts in the frequency, ROA intensity, and sign of each normal mode. As this happens for all of the 55 considered conformations in a unique way, a direct relation between relative differences in geometry and the similarity of the ROA patterns cannot be established. This is supported by reinspecting the list of overlap integrals of the calculated ROA spectra between conformation 1 and the 54 others (Table S7†). The average overlap integrals of the group of conformations that are approximately identical, have less than five, between five and ten, and above ten dihedral angles that are significantly different from conformation 1 are respectively 0.60, 0.38, 0.41, and 0.33. Similar structures give the best spectral overlap with conformation 1. However, it is not so that the overlap integral keeps on dropping with larger geometrical deviations from the reference. This is the result of the complex interplay between the changes in the characteristics of the normal modes upon any geometrical change. This point can be exemplified by the case that has been discussed earlier: the high similarity between the ROA spectrum of the two very different conformations 1 and 50. Somehow, by specific geometrical changes, one can accidentally end up with the same ROA spectrum. Therefore, one should be aware of the fact that for a very diverse and complex compound it is possible that many overlapping normal modes define the ROA intensity, producing accidental matches of very distinct geometries from the actual one with the experiment.

Finally, the isolated aromatic region (1600–1650 cm^−1^) deserves a more profound discussion. As the aromatic rings contribute largely in the ROA intensities, it would be reasonable to believe that their spatial positions dictate the ROA pattern. An attempt to relate the orientations of the five aromatic rings to a certain spectral pattern has been done using Fig. S20 and S21.† First, the conformations were classified according to the orientations of the aromatic rings (through the values of the previously defined dihedral angles 7, 12, 21, 22, 23, 24, 25, and 26; Fig. S20†), and secondly, the ROA spectra have been plotted per group of conformations with identical aromatic ring orientations (Fig. S21†). For none of the groups – even if they seem to persistently trace the same spectral pattern – there is a strict link between the conformation and the spectral pattern. In some cases the ROA pattern is for example merely positive, but those type of patterns are not exclusive for that group, as also merely positive and similar patterns can always be found for one or more structures within another group. This can be rationalized by the fact that even for the isolated aromatic region around 1600–1650 cm^−1^ the peptide cannot be neglected, as normal modes where vibrations are localized both in peptidic and aromatic parts contribute significantly in the aromatic spectral region.

## 4 Conclusions and outlook

In this discourse we expose the limitations of employing the standard ROA approach for determining the conformational behaviour of complex, pharmaceutical compounds. Thereupon, a fresh ROA methodology and analysis strategy has been proposed, leading to a more complete, reliable and transferable study of the system of interest. With this proof-of-concept article we deliver an ROA strategy that can readily by adopted during the study of other challenging cases and we lay the conceptual foundation for a future reliable and high-throughput ROA analysis protocol for the pharmaceutical world.

The strategy that has been put forward is of great interest for all research areas that require a detailed handling of complex conformational landscapes. Using PCA in the way that is presented here is an excellent guide for exploring the conformational space in a geometrically unbiased manner and evaluate the performance of conformational samplings and conformation selection methods. In addition, a general impression of the effect of any kind of geometry optimization becomes available by mapping optimized structures back into the original PC space. For our spectroscopic problem in particular, a PCA analysis succeeded by a *k*-means clustering algorithm has proven to be an adequate preparation for further (ROA) spectral analyses.

As for vancomycin itself, a ROA ensemble – equivalent to the principle behind a NMR ensemble – has been determined. The conformation that is found during NMR experiments is approximated by a purely ROA analysis. Only the orientation of the chlorine atoms on the chlorine-substituted aromatic rings could not be predicted. After estimating the contributions of the peptide, aromatic and sugar entities to the ROA intensities, it has been found that the sugars merely contribute. This breaks with previous studies, where carbohydrates tend to be a prominent contributor in the ROA spectrum. Hence, no conclusions regarding the conformation of the sugar can be drawn from the ROA spectrum. Based upon these results, it appears that the class of glycopeptides forms a unique group of compounds with a specific ROA spectral response. Therefore, we seek to continue the examination of other glycopeptides by means of ROA and the other chiroptical techniques.

## Author contributions

R. A. and J. V. performed experimental measurements, calculations and analysis. R. A. prepared the initial version of the manuscript. W. H. and C. J .supervised the project and the preparation and editing of the manuscript.

## Conflicts of interest

There are no conflicts to declare.

## Supplementary Material

SC-012-D1SC01446C-s001
