# Supplementary material for: Paving the way to conformationally unravel complex glycopeptide antibiotics by means of Raman optical activity
Source: Chem Sci. 2021 Mar 29;12(16):5952–64. doi: 10.1039/d1sc01446c (PMC8867523; doi:10.1039/d1sc01446c)
Supplement: SC-012-D1SC01446C-s001 [file SC-012-D1SC01446C-s001.pdf]

Electronic Supplementary Material (ESI) for Chemical Science.
This journal is © The Royal Society of Chemistry 2021

Supporting Information

Paving the way to conformationally unravel complex glycopeptide antibiotics by means of Raman optical activity

Roy Aerts[†], Jente Vanhove[†], Wouter Herrebout[†] and Christian Johannessen[*†]

† *Department of Chemistry, University of Antwerp, Groenenborgerlaan 171, B-2020 Antwerp, Belgium.*

E-mail: christian.johannessen@uantwerpen.be

Contents

S1 Methodology **3**
S1.1 Conformational analysis . . . 3
S1.2 ROA and Raman spectral calculations . . . 3
S1.3 Experimental Raman and ROA spectroscopy . . . 4

S2 Overlap integral calculations **5**

S3 Normal mode analysis for spectral contributions **6**

S4 Principal component analysis on the conformational ensemble **12**

S5 DFT energies and boltzmann weights **23**

S6 Calculated Raman spectra **24**

S7 Scaling factor determination aromatic region **25**

S8 Spectral ROA overlap integrals **26**

S9 Geometrical features of the 55 doubly optimized representatives **27**

S10 Spectral effect of removing the sugar entity from vancomycin **28**

S11 The aromatic region 30

S1 Methodology

S1.1 Conformational analysis

Six XRD and 160 NMR structures were extracted from the protein data bank (PDB) with entries 1FVM and 1GAC, respectively.[1,2] The XRD PDB file actually contains one supramolecular structure composed of three conventional "back-to-back" vancomycin dimers; each of the six vancomycin structures interacts at the "face" side with a cell-wall precursor analogue (diacetyl-Lys-D-Ala-D-Ala; *nota bene* vancomycin's biological target). The NMR PDB file contains 80 "back-to-back" dimers of the glycopeptide antibiotic A82846B, where each hosts a cell-wall pentapeptide analogue. The assumption of extracting the XRD structures as monomers is validated by the similarity of the core structure of vancomycin between the monomers found in the XRD study and those found in solution.[3,4] The removal of the ligand is valid through the fact that the vancomycin-type glycopeptides form dimers in solution, and lock in the bioactive conformation prior to the eventual complexation with their biological target.[5] For the NMR structures, compound A82846B contains an extra carbohydrate and has one different chiral center in one of the sugar substituents. These were manually modified to form vancomycin monomers. It has been shown that the effect of adding a carbohydrate substituent (and changing one chiral center) does not affect the core conformation of vancomycin, but encourages the dimer formation.[3,5] It is reasonable, therefore, to take these experimental structures for monomers to be added in the pool of conformations that can be adopted by vancomycin. All extracted monomers were prepossessed to contain the same protonation state and a total of 176 atoms. The first of six XRD structure in the 1FVM was used as an input for the conformational programs CONFLEX 7 Rev. A. (reservoir filling algorithm)[6], Pcmodel 10.0 (GMMX search algorithm)[7] and CREST 2.6.1 (metadynamics algorithm combined with genetic Z-matrix crossing)[8]. For CONFLEX 7 the MMFF94 force field[9–13] was selected and the energy cut-off with respect to the lowest energy conformer for the output conformer ensemble was set to 7 kcal/mol. The same force field was used in the Pcmodel 10.0 program, with a final energy threshold of 15 kcal/mol. The *cartesian* and *bond* search options were both used during the sampling -the former is designed to sample locally, whereas the latter is designed to sample throughout the complete potential energy surface. Two different runs were launched for CREST, both with the default parameters in CREST. This program makes use of a semiempiracal quantum mechanics force field. In the first run, the XRD structure as an input was chosen. In the second run, an arbitrary conformation in ensemble, combining the conformations generated by Pcmodel and CONFLEX, that differed significantly from the first input structure, was selected.

S1.2 ROA and Raman spectral calculations

The geometries of the 55 representatives were subjected to two subsequent *ab initio* geometry optimizations (GO) at the B3PW91/6-31G(d,p) and B3PW91/6-31++G(d,p) levels of theory, respectively, with the Gaussian16 rev. A.03 program.[14] The GO at the lower level of theory was necessary to ensure the GO would converge using the 6-31++G(d,p) basis set. All the optimized geometries were determined to be located in a minimum on the potential energy surface using the Hessian calculated on the corresponding level of theory. For spectral

computations, the Hessian, Raman- and ROA tensors were calculated using the B3PW91/6-31++G(d,p) level of theory. The harmonic frequencies were rescaled by multiplying by 0.987, unless stated otherwise, to compensate for the overestimation due to the harmonic approximation and used basis set. This scaling factor has previously been determined by Mensch *et al.*[15] The Raman and ROA intensities were calculated according to the experimental setup, with an excitation wavelength of 532 nm. A temperature correction (298 K) was applied on all Raman and ROA intensities, followed by a Lorentzian line broadening with a full width at half maximum (FWHM) of 20 cm^{-1}.[16,17] During all the *ab initio* calculations the integral equation formalism model (IEFPCM) as implemented in Gaussian16 was used to implicitly account for the water solvent, with a dielectric constant of $\epsilon = 78.3553$. For spectral comparisons the overlap integral as calculated by Mensch *et al.* was used (mathematical details in section S2).[15]

S1.3 Experimental Raman and ROA spectroscopy

Vancoymcin has been purchased from AmBeed and has been used without further purification. The sample was prepared using an acetate buffer to adjust the pH to 3.6, in accordance with the sample examined by NMR -the pH must be lower than 4 for vancomycin to be soluble in aqueous solution.[2] The concentration of the sample was 50 mg/mL. All Raman and ROA spectra were recorded under ambient conditions using the ChiralRAMAN-2X scattered circular polarization (SCP) ROA instrument (BioTools, Inc.).[18] The intensity of the right- and left circularly polarized Raman scattered photons (I_R and I_L) are detected simultaneously. The Raman and ROA spectra are obtained by adding ($I_R + I_L$) or subtracting ($I_R - I_L$) the two intensities, respectively. The laser excitation wavelength of the instrument is 532 nm. The power of the laser at the source was 800 mW. The total measurement time was 48 hours; the illumination time single scan was set to 2.2 seconds. A solvent spectrum has been subtracted from the Raman spectrum, and a subsequent baseline correction procedure, as reported by Boelens *et al.*, was applied.[19] The ROA spectrum has been smoothed using a third-order nine-points Savitzky-Golay filter.

S2 Overlap integral calculations

All the overlap integrals reported in this manuscript are calculated according to the following formula[15]:

$$S_{fg} = \frac{\int f(\sigma\tilde{v})g(\tilde{v})}{\sqrt{\int f(\sigma\tilde{v})^2 d\tilde{v} \int g(\tilde{v})^2 d\tilde{v}}} \quad (1)$$

Where f and g represent the theoretical and calculated spectra, σ the scaling factor for the wavenumbers of the calculated spectrum, and $\tilde{v}$ the wavenumber. The overlap integral varies between 0 and 1 for Raman and -1 and 1 for ROA spectral comparisons.

The overlap integral between the ROA experimental and calculated spectra is typically used to evaluate the spectral match. When exact value an overlap integral must take to be considered as a satisfying match has been a matter of debate within the chiroptical community. The rule of thumb is that if a value is equal or higher than 0.4 - 0.6, then the spectral match is satisfying and should be considered in an analysis.[20,21] However, it should be noted that these values are highly dependent on the compound, and very often -as we do for vancomycin- one should interpret the overlap integral values in a relative way. The protocol used here to compare the calculated spectra to the experimental one is (1) take the maximum overlap integral (0.53; see main text or Table S7), (2) assume that all spectra that are within 0.10 equally match the experiment (this to prevent the overvaluation of the exact absolute number).

S3 Normal mode analysis for spectral contributions

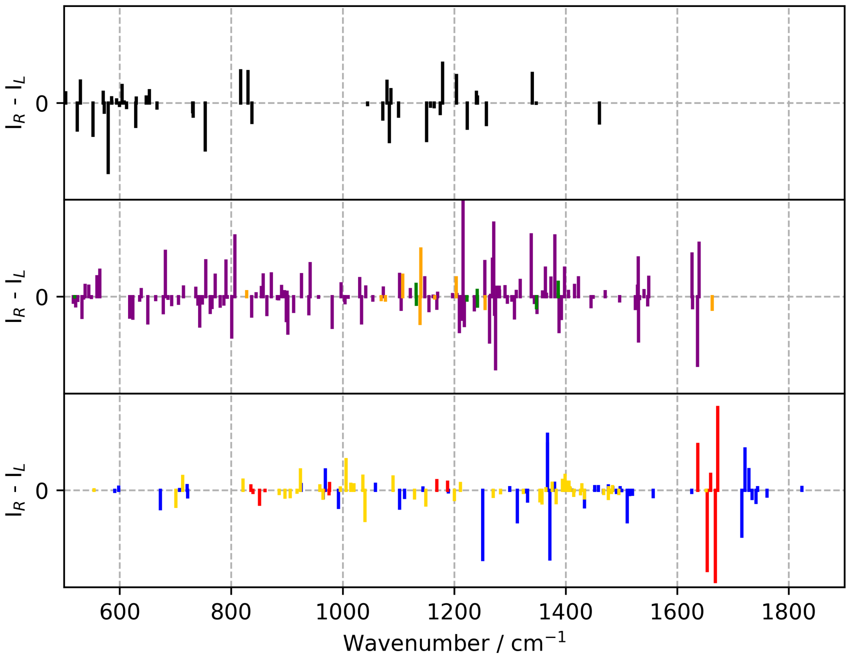

Fig. S1 The assignment of each normal mode of conformation 1 (with their corresponding Raman optical activity intensity) to one of the following categories: peptide+aromat+sugar (black; top), peptide+aromat (purple; middle), peptide+sugar (green; middle), aromat+sugar (orange; middle), peptide (blue; bottom), aromat (red; bottom), sugar (yellow; bottom). See at the methodology section in the main text for the assignment. No scaling factor was applied to the normal mode wavenumbers.

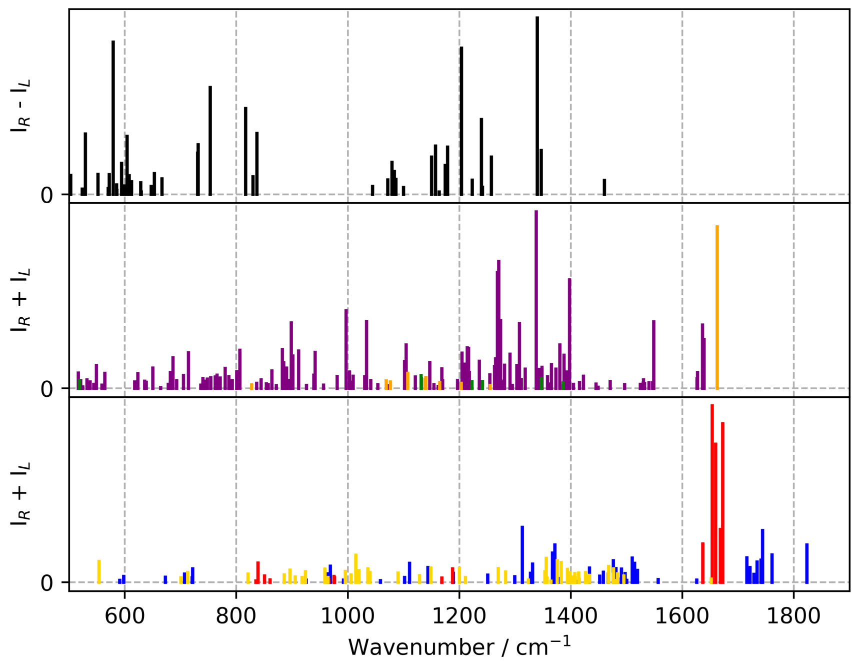

Fig. S2 The assignment of each normal mode of conformation 1 (with their corresponding Raman intensity) to one of the following categories: peptide+aromat+sugar (black; top), peptide+aromat (purple; middle), peptide+sugar (green; middle), aromat+sugar (orange; middle), peptide (blue; bottom), aromat (red; bottom), sugar (yellow; bottom). See at the methodology section in the main text for the assignment. No scaling factor was applied to the normal mode wavenumbers.

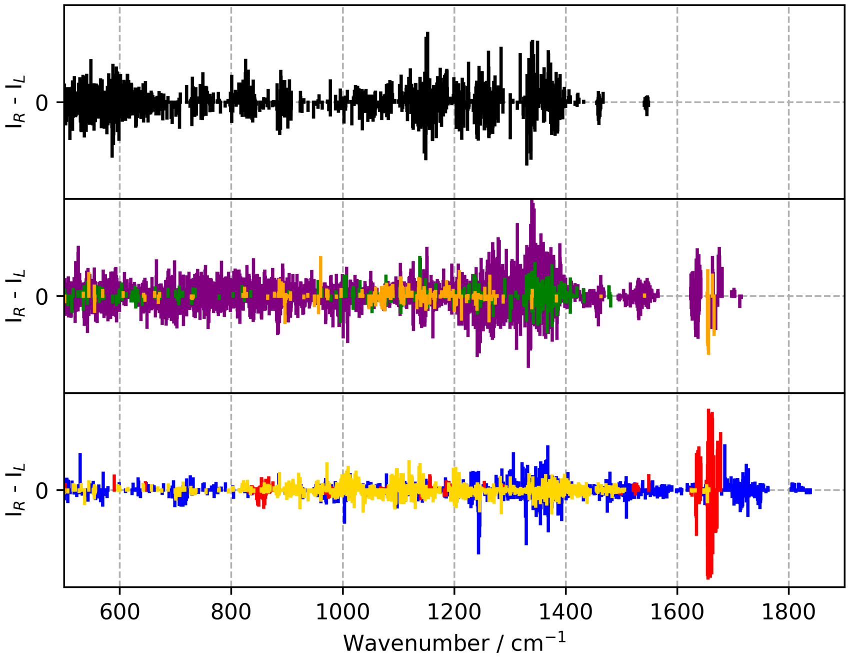

Fig. S3 The assignment of each normal mode of all 55 conformations (with their corresponding Raman optical activity intensity) to one of the following categories: peptide+aromat+sugar (black; top), peptide+aromat (purple; middle), peptide+sugar (green; middle), aromat+sugar (orange; middle), peptide (blue; bottom), aromat (red; bottom), sugar (yellow; bottom). See at the methodology section in the main text for the assignment. No scaling factor was applied to the normal mode wavenumbers.

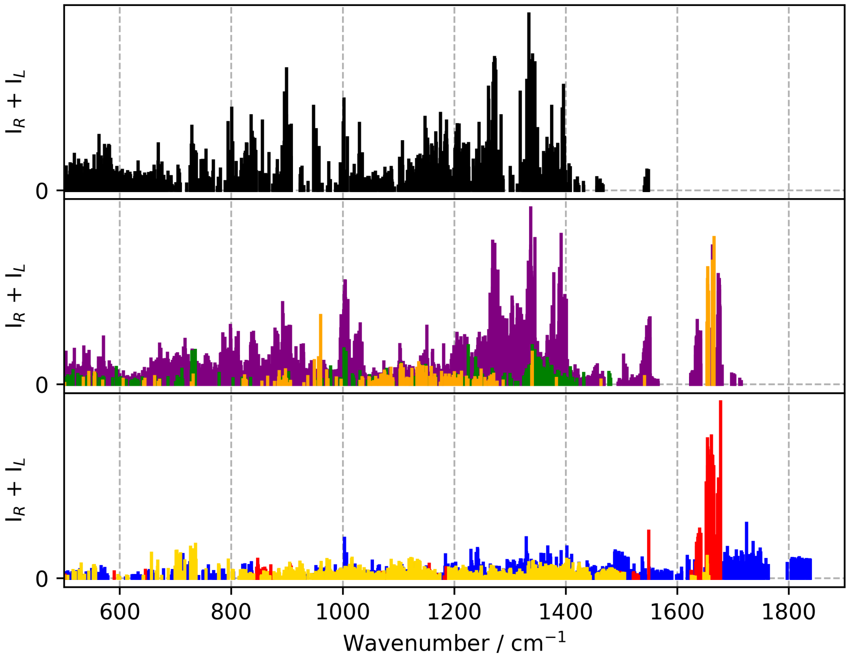

Fig. S4 The assignment of each normal mode of all 55 conformations (with their corresponding Raman intensity) to one of the following categories: peptide+aromat+sugar (black; top), peptide+aromat (purple; middle), peptide+sugar (green; middle), aromat+sugar (orange; middle), peptide (blue; bottom), aromat (red; bottom), sugar (yellow; bottom). See at the methodology section in the main text for the assignment. No scaling factor was applied to the normal mode wavenumbers.

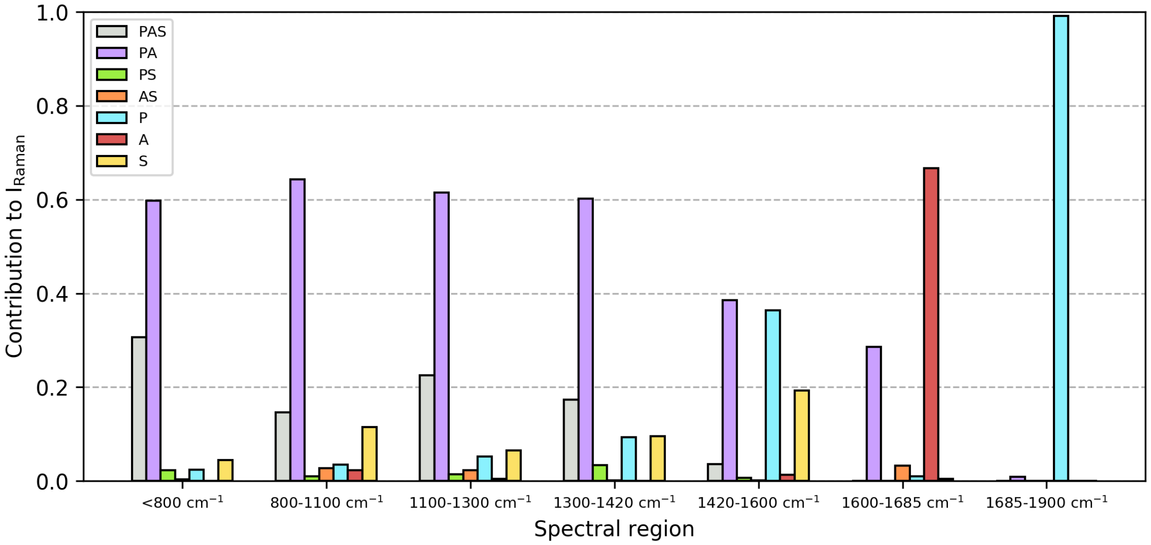

Fig. S5 A bar graph to visualize the contributions of the normal modes -assigned to the peptide (P), sugar (S) and aromatic (A) entities, or any combination thereof (PAS, PA, PS, AS)- to the total Raman intensity in the specified spectral regions. The percentage is always calculated with respect to the total Raman intensity in that spectral region. The wavenumbers have not been scaled during this analysis.

Table S1 The average number of normal modes in the specified regions assigned to vibrations originating from the peptide (P), aromatic (A) and sugar (S) entities, or any combination thereof (PAS, PA, PS, AS). The average has been taken over all 55 conformations.

Chemical entity	Sign	<800 cm^{-1}	800-1100	1100-1300	1300-1420	1420-1600	1600-1685	1685-1900
PAS	+	12 (18%)	4 (6%)	7 (10%)	2 (4%)	1 (3%)	0 (0%)	0 (0%)
	-	11 (17%)	4 (6%)	6 (9%)	2 (4%)	1 (3%)	0 (0%)	0 (0%)
PA	+	17 (26%)	17 (25%)	16 (25%)	10 (21%)	6 (16%)	3 (23%)	0 (0%)
	-	19 (29%)	18 (26%)	14 (22%)	6 (13%)	6 (16%)	2 (15%)	0 (0%)
PS	+	1 (2%)	1 (1%)	1 (2%)	1 (2%)	0 (0%)	0 (0%)	0 (0%)
	-	1 (2%)	0 (0%)	1 (2%)	1 (2%)	0 (0%)	0 (0%)	0 (0%)
AS	+	0 (0%)	1 (1%)	1 (2%)	0 (0%)	0 (0%)	0 (0%)	0 (0%)
	-	0 (0%)	1 (1%)	1 (2%)	0 (0%)	0 (0%)	0 (0%)	0 (0%)
P	+	1 (2%)	3 (4%)	3 (5%)	5 (11%)	7 (19%)	1 (8%)	4 (50%)
	-	1 (2%)	2 (3%)	4 (6%)	5 (11%)	7 (19%)	1 (8%)	4 (50%)
A	+	0 (0%)	2 (3%)	1 (2%)	0 (0%)	0 (0%)	2 (15%)	0 (0%)
	-	0 (0%)	3 (4%)	0 (0%)	0 (0%)	0 (0%)	3 (23%)	0 (0%)
S	+	1 (2%)	7 (10%)	4 (6%)	8 (17%)	4 (11%)	0 (0%)	0 (0%)
	-	1 (2%)	6 (9%)	5 (8%)	7 (15%)	5 (14%)	1 (8%)	0 (0%)
Total	+	32 (49%)	35 (51%)	33 (52%)	26 (55%)	18 (49%)	6 (46%)	4 (50%)
	-	33 (51%)	34 (49%)	31 (48%)	21 (45%)	19 (51%)	7 (54%)	4 (50%)
**Total**		**65 (100%)**	**69 (100%)**	**64 (100%)**	**47 (100%)**	**37 (100%)**	**13 (100%)**	**8 (100%)**

Table S2 An estimation of the percentual contribution of one normal mode in the specified regions assigned to a certain category: peptide (P), aromatic (A) and sugar (S) entities, or any combination thereof (PAS, PA, PS, AS). These percentage are calculated by normalizing the values in Fig. 9 in the main text with the amount of normal modes in Table S1. This percentage has not been calculated when the values of Table S1 are zero.

Chemical entity	Sign	<800 cm^{-1}	800-1100	1100-1300	1300-1420	1420-1600	1600-1685	1685-1900
PAS	+	1.5%	2.1%	2.0%	4.1%	1.9%	-	-
	-	1.7%	1.9%	2.4%	3.3%	5.7%	-	-
PA	+	1.6%	1.7%	1.7%	3.7%	4.0%	8.5%	-
	-	1.5%	1.6%	1.8%	2.8%	3.9%	5.6%	-
PS	+	1.0%	1.0%	1.3%	2.7%	-	-	-
	-	0.9%	-	0.7%	1.2%	-	-	-
AS	+	-	1.9%	1.8%	-	-	-	-
	-	-	1.7%	1.0%	-	-	-	-
P	+	1.5%	0.7%	0.9%	1.6%	1.8%	0.5%	12.1%
	-	1.5%	1.0%	0.8%	1.3%	2.4%	0.4%	12.4%
A	+	-	0.6%	0.5%	-	-	13.6%	-
	-	-	0.5%	-	-	-	9.1%	-
S	+	0.5%	1.2%	0.8%	0.7%	1.3%	-	-
	-	0.7%	1.0%	0.8%	0.8%	1.5%	0.4%	-

S4 Principal component analysis on the conformational ensemble

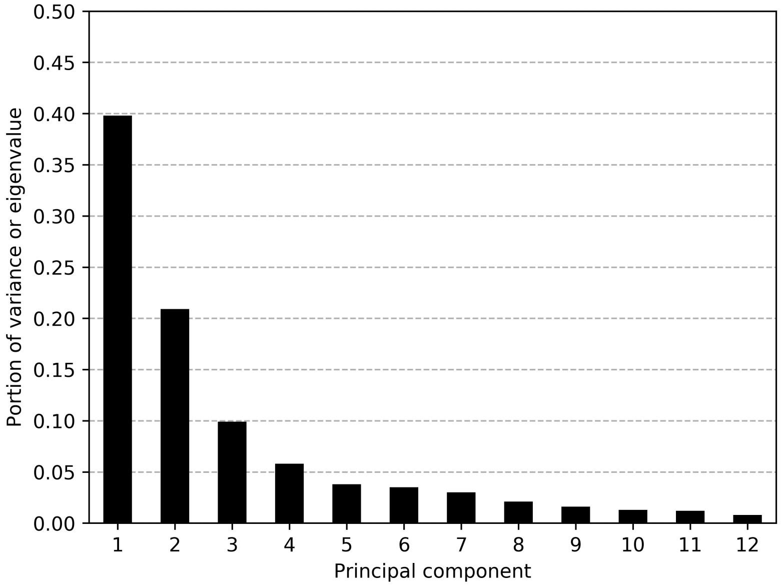

Fig. S6 The scree plot of the principal component analysis on the geometries, indicating the portion of the variance (or eigenvalue) that is represented by each principal component (PC). Only the PCs representing a portion of the variance above 1% are included.

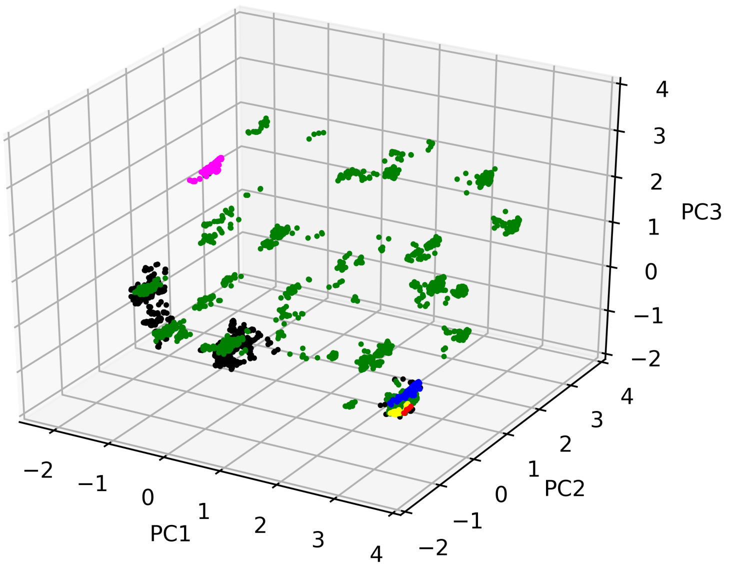

Fig. S7 The 3D principal component scores plot after applying the principal component analysis on the geometries. The colour indicates the origin of the conformation: XRD (red; 6 conformations), NMR (yellow; 160 conformations), Pcmodel (green; 3 452 conformations), CONFLEX (black; 7 768 conformation), CREST-1 (blue; 519 conformations), CREST-2 (magenta; 472 conformations).

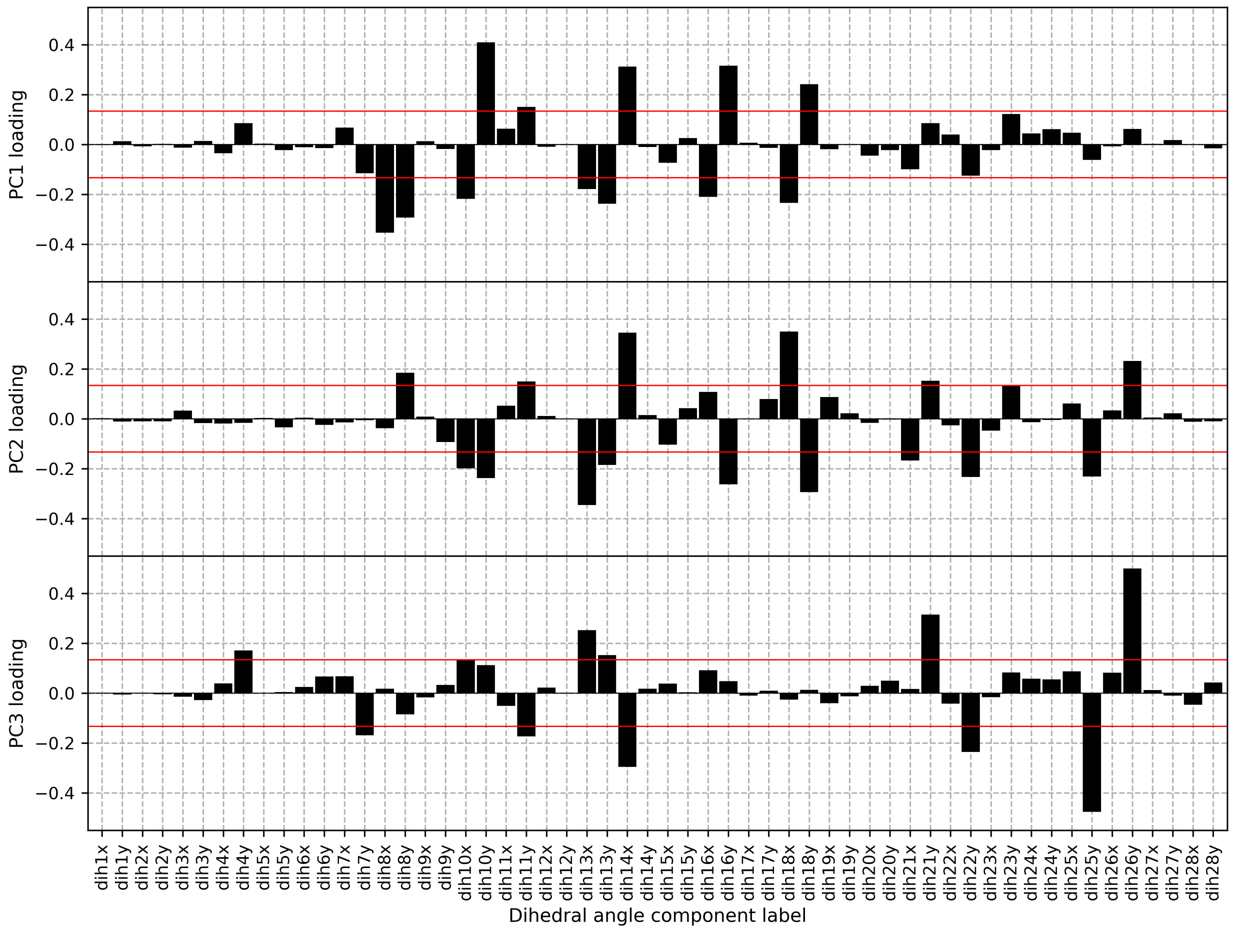

Fig. S8 The principal component loadings for each variable of the first three principal components. If a bar exceeds the red threshold, the corresponding variable contributes more to the linear combination of that particular principal component than when all variables were to contribute equally.

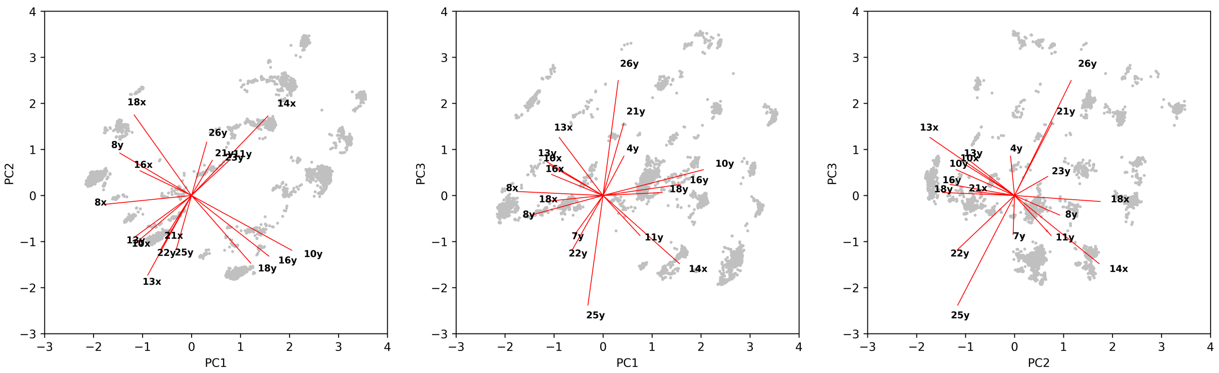

Fig. S9 The principal component biplot, visualizing the principal component loadings as vectors on top of the 2D projections of the 3D PC scores plot. In essence a visual representation of Fig. S8.

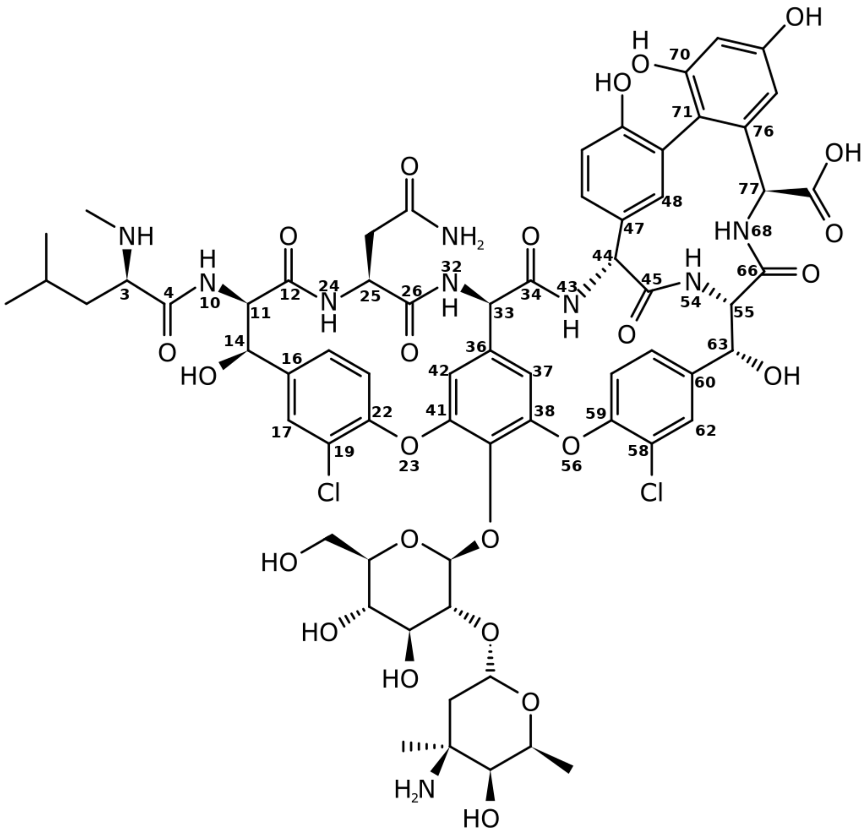

Fig. S10 The chemical structure of vancomycin with the atom numbers that are used for calculating the dihedral angles. The labels is always the non-hydrogen atom.

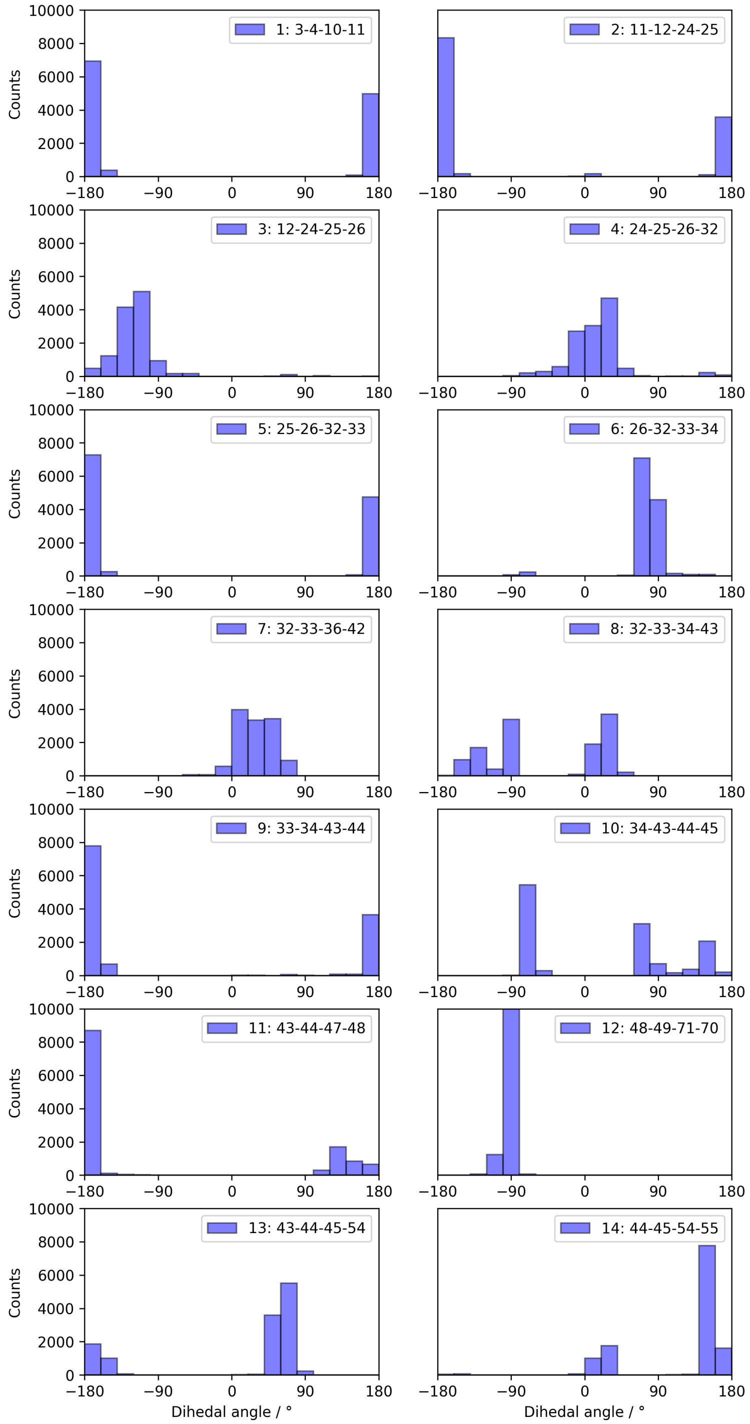

Fig. S11 The dihedral angle distribution of the first 14 dihedral angles used in the principal component analysis for the 12 337 conformations. The legend indicates what dihedral angle is considered (before the colon; labels from Fig. 2 in the main text) and what atoms were used (four atom numbers after the colon; labes from Fig. S10).

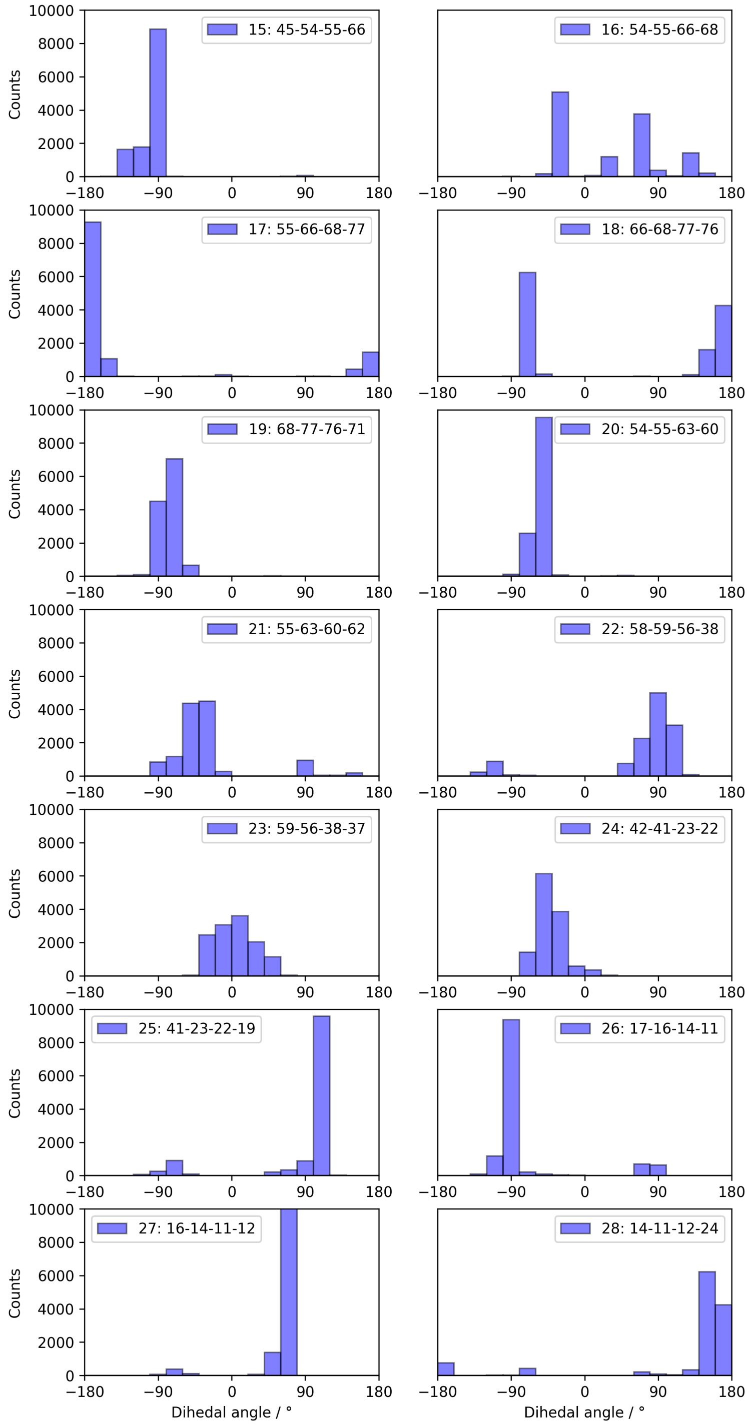

Fig. S12 The dihedral angle distribution of the last 14 dihedral angles used in the principal component analysis for the 12 337 conformations. The legend indicates what dihedral angle is considered (before the colon; labels from Fig. 2 in the main text) and what atoms were used (four atom numbers after the colon; labels from Fig. S10).

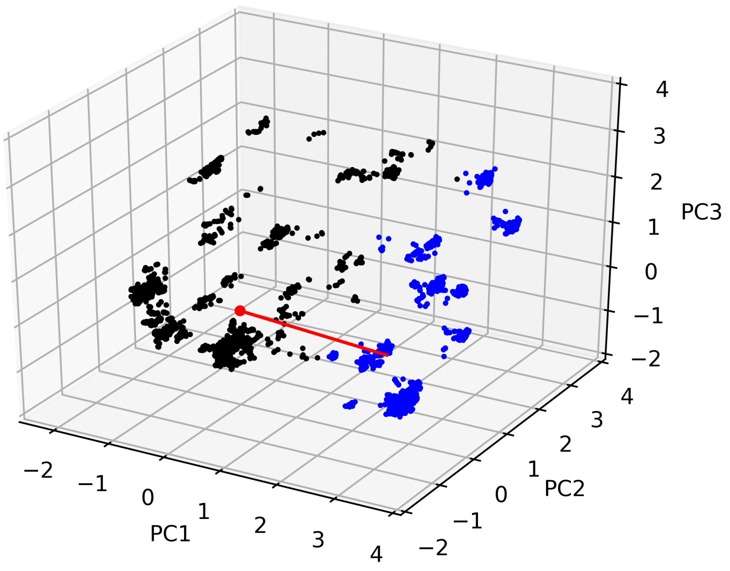

Fig. S13 The 3D scores plot where all *trans* and *cis* configurated conformations are indicated with a black and blue colours, respectively. The x-, y-, and z-components of the red vector are the PC loadings of the x-component of dihedral angle 14 (Fig. 2, main text) for the first three principal components.

Table S3 The amount of conformations that contain a *cis* peptidic bond per ω dihedral angle and per origin of that conformation. For the dihedral angles 1 and 3 no *cis* configurations were present. The dihedral angle number as indicated in Fig. 2 in the main text corresponding to the concerned ω-angle is given as a number between brackets in the column ω-angle. The two percentages underneath the amount of *cis* configurated peptidic bonds (in bold) represent the percentage of *cis* configured conformations with respect to the total conformations arising from the same origin (left) and the total amount of conformations (12 377; right).

**ω-angle**	**CONFLEX**	**Pcmodel**	**CREST 1**	**CREST 2**	**NMR**	**XRD**
**2**	**192**	0	0	0	0	0
(2)	2.47 % ; 1.55 %	0	0	0	0	0
**4**	**12**	**79**	0	0	0	0
(9)	0.64 % ; 0.10 %	2.29 % ; 0.15 %	0	0	0	0
**5**	**187**	**1965**	**519**	0	**160**	**6**
(14)	2.41 % ; 1.51 %	56.92 % ; 15.88 %	100 % ; 4.19 %	0	100 % ; 1.29 %	100 % ; 0.05 %
**6**	**116**	**15**	0	0	0	0
(17)	1.49 % ; 0.94 %	0.43 % ; 0.12 %	0	0	0	0

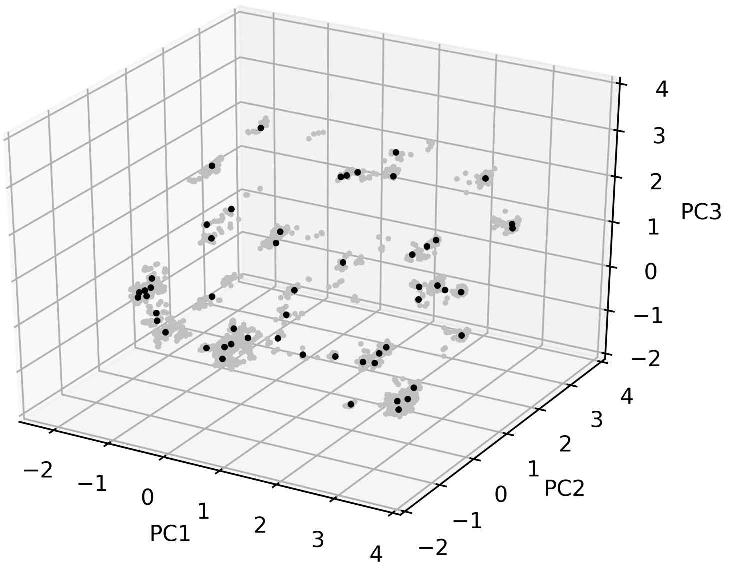

Fig. S14 The 3D scores plot where the 55 selected representative conformations by the k-means clustering algorithm are indicated in black. All 12 377 conformations are depicted in grey.

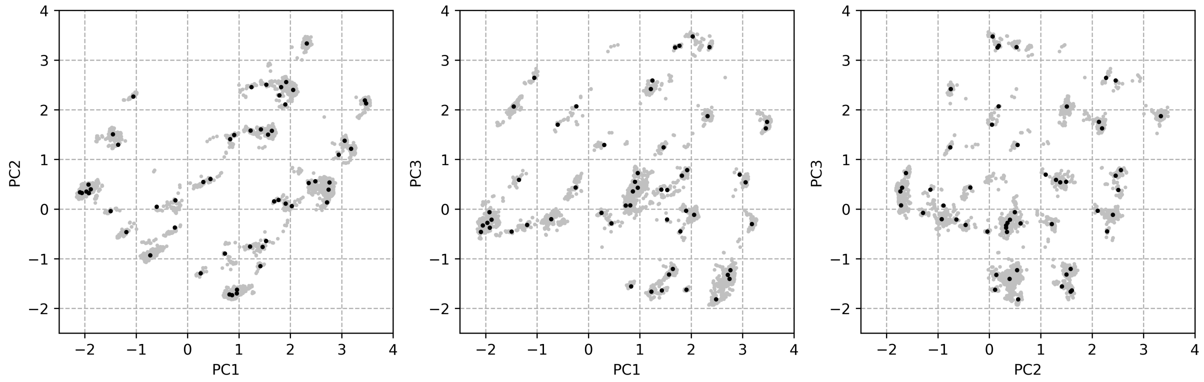

Fig. S15 The 2D projections of the 3D scores plot where the 55 selected representative conformations by the k-means clustering algorithm are indicated in black. All 12 377 conformations are depicted in grey.

Table S4 The distances in the 3D PC scores space a *travelled* after subjecting each of the 55 representatives to the two subsequent geometry optimizations (GO; columns 4-6); all in arbitrary units. The angle between the two vectors corresponding to these changes are given in column 7.

Conformation	Origin	Reference No.	*d*(Start-GO1)	*d*(GO1-GO2)	*d*(Start-GO2)	∠(GO1-GO2) / °
1	NMR	164	0.32	0.06	0.28	47
2	Pcmodel	1219	0.26	0.04	0.28	111
3	Pcmodel	1482	0.29	0.03	0.27	49
4	Pcmodel	1584	0.20	0.02	0.21	127
5	Pcmodel	1651	0.43	0.06	0.42	77
6	Pcmodel	1662	0.31	0.03	0.31	78
7	Pcmodel	1741	0.22	0.08	0.25	101
8	Pcmodel	1822	0.20	0.03	0.21	117
9	Pcmodel	1904	0.26	0.02	0.27	130
10	Pcmodel	1931	0.13	0.04	0.11	56
11	Pcmodel	2039	0.07	0.05	0.08	90
12	Pcmodel	2155	0.40	0.14	0.52	143
13	Pcmodel	2159	0.60	0.07	0.62	104
14	Pcmodel	2171	0.21	0.04	0.25	148
15	Pcmodel	2198	0.26	0.15	0.19	49
16	Pcmodel	2350	0.41	0.13	0.54	162
17	Pcmodel	2383	0.33	0.05	0.30	63
18	Pcmodel	2495	0.35	0.06	0.30	29
19	Pcmodel	2547	0.27	0.04	0.23	24
20	Pcmodel	2761	0.11	0.02	0.09	23
21	Pcmodel	2852	0.30	0.01	0.31	132
22	Pcmodel	2862	0.07	0.03	0.09	115
23	Pcmodel	2883	0.40	0.07	0.38	68
24	Pcmodel	2979	0.54	0.03	0.56	125
25	Pcmodel	3106	0.22	0.06	0.26	128
26	Pcmodel	3140	0.13	0.22	0.30	115
27	Pcmodel	3290	0.04	0.04	0.06	114
28	Pcmodel	3303	0.38	0.06	0.35	55
29	Pcmodel	3342	0.39	0.02	0.41	147
30	Pcmodel	3596	0.11	0.02	0.13	139
31	Pcmodel	392	0.67	0.02	0.67	91
32	Pcmodel	468	0.26	0.03	0.28	126
33	Pcmodel	585	0.23	0.09	0.26	98
34	Pcmodel	664	0.45	0.05	0.41	48
35	Pcmodel	755	0.28	0.03	0.28	89
36	Pcmodel	764	0.41	0.10	0.50	149
37	Pcmodel	850	0.19	0.06	0.14	24
38	Pcmodel	858	0.30	0.02	0.31	127
39	Pcmodel	888	0.25	0.08	0.26	94
40	Pcmodel	980	0.66	0.06	0.72	165
41	CONFLEX	10366	0.56	0.10	0.65	165
42	CONFLEX	10397	0.21	0.31	0.13	20
43	CONFLEX	10569	0.22	0.06	0.17	5
44	CONFLEX	10623	0.69	0.22	0.83	122
45	CONFLEX	10902	0.33	0.03	0.31	67
46	CONFLEX	10959	0.74	0.18	0.60	34
47	CONFLEX	11081	0.12	0.29	0.33	99
48	CONFLEX	4052	0.32	0.06	0.26	13
49	CONFLEX	4544	0.14	0.06	0.11	42
50	CONFLEX	5600	0.52	0.06	0.57	132
51	CONFLEX	8226	0.20	0.12	0.09	17
52	CONFLEX	9476	0.14	0.02	0.13	57
53	CONFLEX	9824	0.24	0.06	0.19	30
54	CREST-1	11556	0.27	0.03	0.30	168
55	CREST-2	11914	0.32	0.05	0.36	137

S5 DFT energies and boltzmann weights

Table S5 The relative enthalpies and corresponding Boltzmann weights of the conformations that contribute more than 0.5% to the Boltzmann weighted spectrum.

**Conformation**	H° / **kcal.mol^{-1}**	ΔH° / **kcal.mol^{-1}**	**Boltzmann weight / %**
31	-3624255.82	0.00	92.0
50	-3624254.29	1.53	7.0
38	-3624252.97	2.85	0.8

S6 Calculated Raman spectra

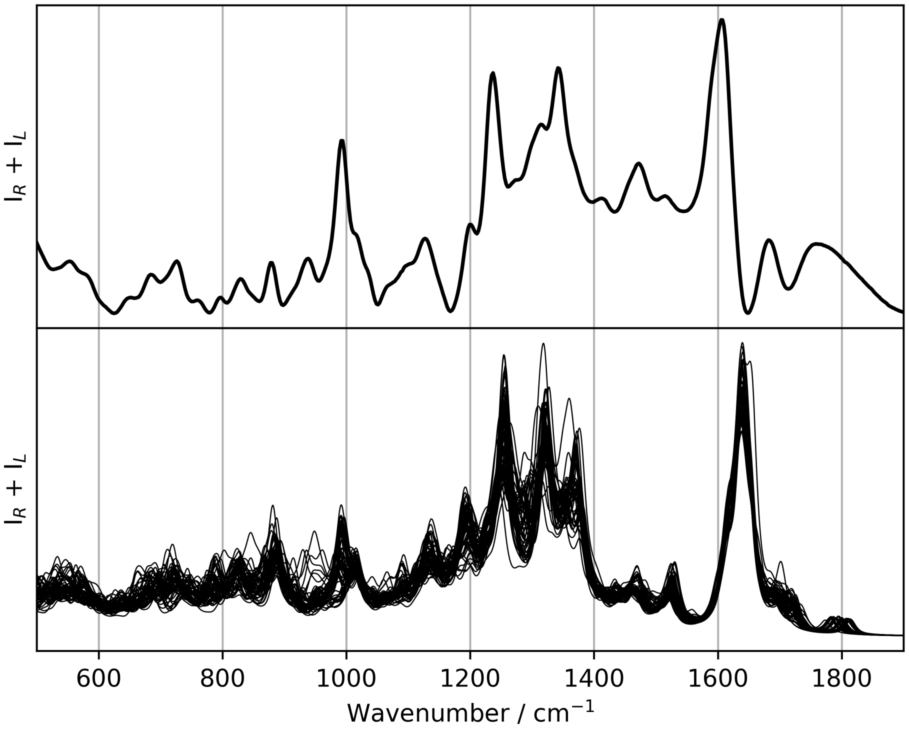

Fig. S16 The Raman spectra of vancomycin: the experimental recording in aqueous solution (top) and the calculated spectra of the 55 representative conformations (bottom) after applying the scaling factor of 0.987).

S7 Scaling factor determination aromatic region

Table S6 The average overlap integral (S_{fg}; using equation 1) of all 55 Raman spectra in the 1550-1650 cm^{-1} spectral region when applying a scaling factor between 0.960 and 0.970. The scaling factor that maximizes the overlap integral is indicated in bold.

Scaling factor	S_{fg}(Ram, 1550-1650 cm^{-1})
0.960	0.9485
0.961	0.9594
0.962	0.9680
0.963	0.9742
0.964	0.9776
**0.965**	**0.9781**
0.966	0.9755
0.967	0.9699
0.968	0.9609
0.969	0.9487
0.970	0.9333

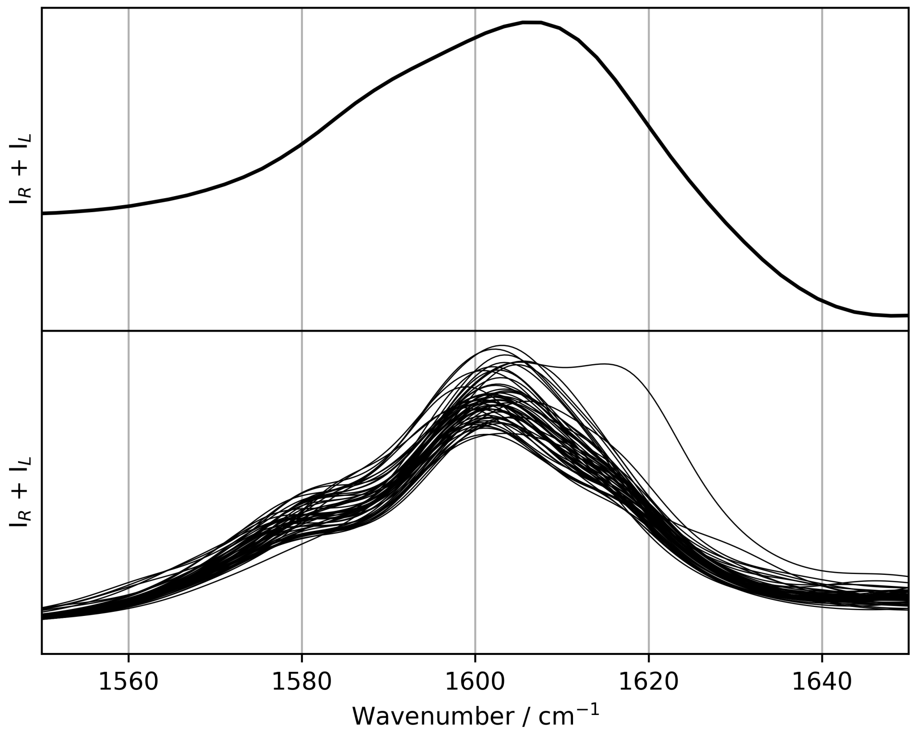

Fig. S17 The Raman spectra of vancomycin: the experimental recording in aqueous solution (top) and the calculated spectra of the 55 representative conformations (bottom) after applying the scaling factor of 0.965 (Table S6). These are zoomed in versions (apart from the scaling factor used for the calculated Raman spectra) of figure S16.

S8 Spectral ROA overlap integrals

Table S7 The overlap integrals of all calculated ROA spectra in the regions 500-1900 cm^{-1} (and Raman), 500-1420 cm^{-1} (reg1), and 1550-1650 cm^{-1} (reg2; scaling factor 0.965, see Table S6) with respect to conformation 1 (NMR structure) and the experimental spectrum. In column 6 the Eucledian 3D PC scores distances with respect to conformation 1 are given.

Conf.	S_{fg}(ROA)	S_{fg}(ROA:reg1)	S_{fg}(ROA:reg2)	S_{fg}(Ram)	PCA dist HB	S_{fg}(ROA:exp)	S_{fg}(ROA:reg1)	S_{fg}(ROA:reg2)	S_{fg}(Ram)
1	1.00	1.00	1.00	1.00	0.00	0.48	0.55	0.03	0.76
2	0.57	0.66	-0.28	0.99	2.32	0.52	0.61	-0.07	0.77
3	0.21	0.23	0.14	0.97	4.78	0.20	0.27	0.42	0.77
4	0.45	0.53	-0.37	0.98	3.21	0.37	0.43	0.18	0.76
5	0.05	0.06	0.05	0.98	4.39	0.06	0.10	-0.06	0.75
6	0.46	0.48	0.53	0.98	2.86	0.22	0.29	0.52	0.78
7	0.41	0.43	0.22	0.98	3.35	0.19	0.22	0.49	0.76
8	0.35	0.50	-0.46	0.99	3.74	0.46	0.58	0.11	0.77
9	0.45	0.52	-0.19	0.99	1.68	0.27	0.32	0.35	0.75
10	0.22	0.24	0.34	0.98	5.95	0.09	0.12	-0.36	0.75
11	0.51	0.65	-0.46	0.99	1.60	0.53	0.65	0.24	0.77
12	0.39	0.38	0.82	0.98	4.74	0.35	0.41	0.36	0.77
13	0.17	0.16	0.38	0.98	4.44	0.18	0.21	0.01	0.77
14	0.25	0.48	-0.58	0.99	4.51	0.19	0.29	0.19	0.75
15	0.35	0.41	-0.16	0.99	3.16	0.29	0.34	-0.29	0.76
16	0.53	0.48	0.94	0.99	3.95	0.33	0.44	0.18	0.77
17	0.35	0.43	-0.37	0.98	4.77	0.27	0.33	0.62	0.78
18	0.26	0.41	-0.56	0.98	2.59	0.34	0.44	0.24	0.76
19	0.48	0.54	-0.25	0.99	1.73	0.41	0.47	0.52	0.76
20	0.57	0.60	0.36	0.99	1.65	0.30	0.36	-0.27	0.75
21	0.53	0.56	-0.09	0.99	0.99	0.30	0.34	-0.08	0.77
22	0.66	0.67	0.56	0.98	0.17	0.39	0.46	0.49	0.75
23	0.47	0.47	0.52	0.98	2.80	0.19	0.24	0.69	0.75
24	0.49	0.44	0.95	0.98	1.52	0.35	0.41	0.26	0.78
25	0.33	0.41	-0.38	0.98	2.55	0.35	0.40	0.17	0.76
26	0.38	0.47	-0.52	0.99	3.15	0.40	0.46	0.07	0.76
27	0.33	0.31	0.66	0.98	3.20	0.33	0.39	0.31	0.76
28	0.39	0.48	-0.38	0.98	2.59	0.33	0.41	0.25	0.77
29	0.15	0.20	0.11	0.97	2.38	0.13	0.27	-0.04	0.75
30	0.47	0.55	-0.41	0.99	2.32	0.33	0.39	0.30	0.75
31	0.20	0.21	0.18	0.99	4.46	0.07	0.09	-0.03	0.78
32	0.14	0.22	-0.20	0.98	4.43	0.16	0.22	-0.11	0.76
33	0.56	0.52	0.96	0.98	2.36	0.17	0.22	0.12	0.77
34	0.12	0.23	-0.55	0.98	3.37	0.16	0.21	0.30	0.75
35	0.18	0.40	-0.67	0.99	3.56	0.25	0.37	0.18	0.76
36	0.44	0.45	0.54	0.99	2.89	0.22	0.26	0.72	0.76
37	0.31	0.27	0.91	0.97	4.65	-0.01	0.02	0.05	0.76
38	0.51	0.56	0.00	0.99	1.72	0.39	0.45	0.39	0.77
39	0.21	0.29	-0.39	0.98	3.60	0.17	0.20	0.04	0.76
40	0.49	0.42	0.93	0.99	2.65	0.25	0.32	0.22	0.77
41	0.39	0.31	0.87	0.98	2.60	0.21	0.29	0.25	0.76
42	0.51	0.45	0.90	0.97	4.78	0.08	0.11	0.04	0.76
43	0.26	0.17	0.94	0.97	4.74	-0.08	-0.08	-0.14	0.75
44	0.43	0.39	0.68	0.98	2.71	0.13	0.17	0.36	0.76
45	0.76	0.77	0.82	0.99	0.13	0.51	0.57	0.19	0.76
46	0.42	0.39	0.73	0.97	4.71	0.11	0.16	-0.34	0.76
47	0.49	0.46	0.84	0.97	2.50	0.17	0.22	0.30	0.77
48	0.29	0.26	0.61	0.98	4.71	0.10	0.14	-0.34	0.77
49	0.40	0.34	0.99	0.98	4.71	0.11	0.13	0.04	0.76
50	0.62	0.61	0.82	0.99	2.82	0.35	0.43	0.36	0.76
51	0.51	0.53	0.59	0.96	4.11	0.20	0.27	0.17	0.74
52	0.44	0.39	0.97	0.98	4.69	0.15	0.19	-0.10	0.76
53	0.38	0.31	0.97	0.97	4.71	0.05	0.08	0.11	0.76
54	0.73	0.74	0.66	0.99	0.17	0.47	0.55	0.36	0.77
55	0.30	0.30	0.38	0.96	5.49	0.14	0.19	-0.18	0.73

S9 Geometrical features of the 55 doubly optimized representatives

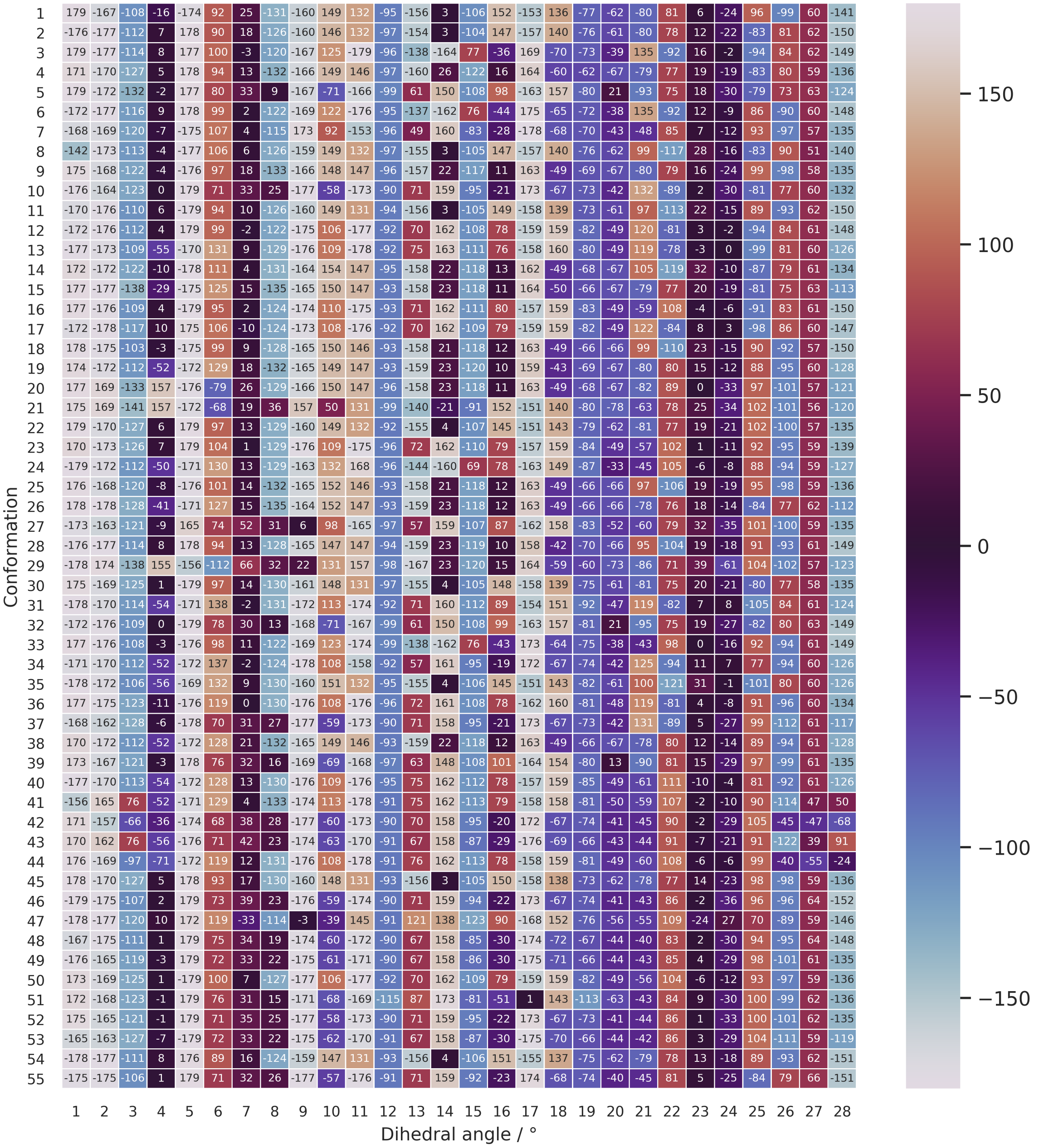

Fig. S18 A heatmap representation of the geometries behind the 55 conformations for all 28 dihedral angles (Fig. 2; main text).

S10 Spectral effect of removing the sugar entity from vancomycin

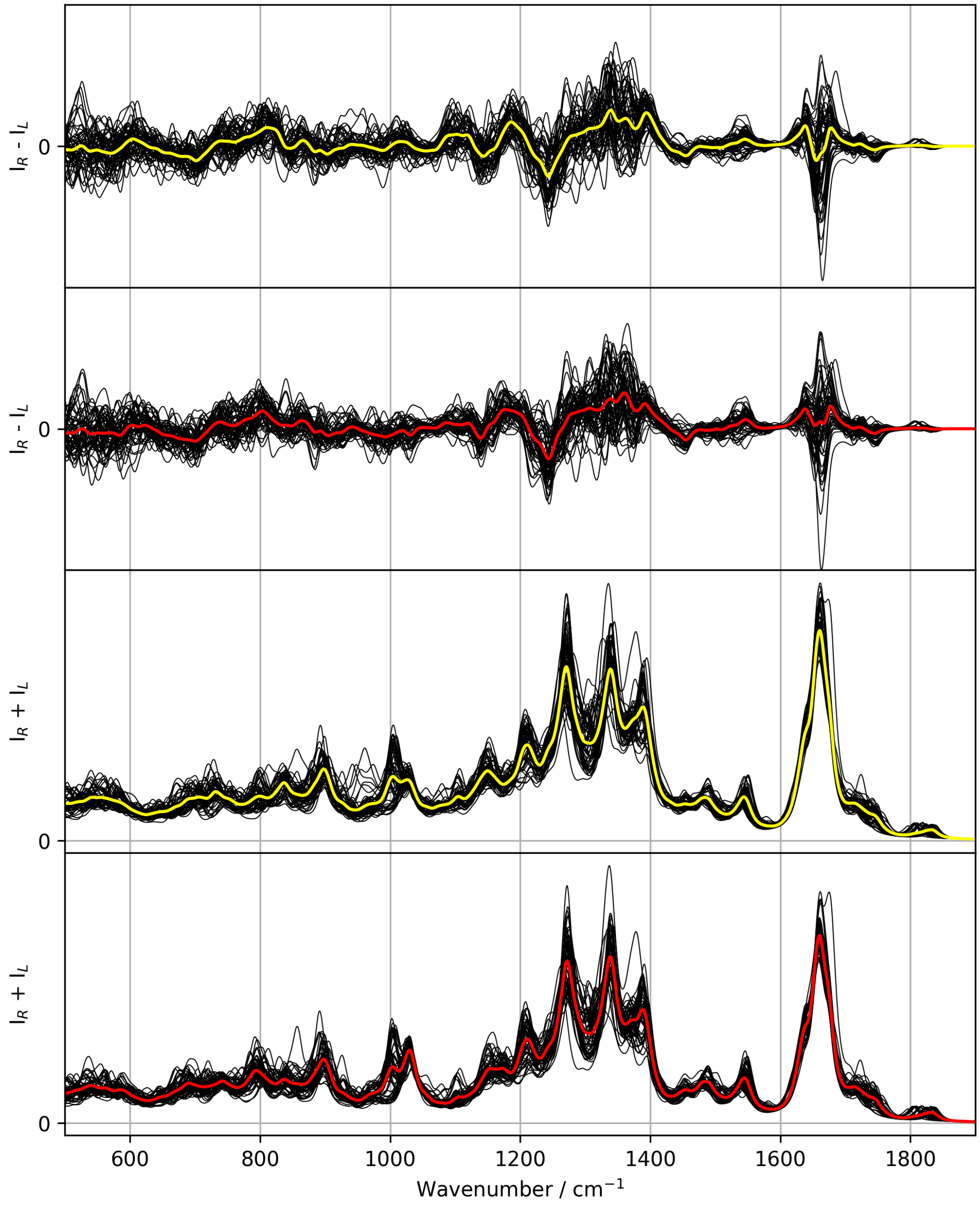

Fig. S19 The ROA (two top) and Raman (two bottom) of the 55 conformations of vancomycin (black; their average in yellow and red) with (panels 1 and 3) and without (panels 2 and 4) the sugar entities.

Table S8 The overlap integrals between the ROA and Raman spectra (500-1900 cm^{-1}; see Fig. S19) of vancomycin with and without the sugar moieties, for each of the 55 conformations.

Conformation	S_{fg}(ROA)	S_{fg}(Raman)
1	0.80	0.99
2	0.86	0.99
3	0.86	0.99
4	0.89	0.99
5	0.93	0.99
6	0.84	0.99
7	0.92	0.99
8	0.89	0.99
9	0.94	0.99
10	0.85	0.99
11	0.88	0.99
12	0.87	0.99
13	0.90	0.99
14	0.93	0.99
15	0.90	0.99
16	0.82	1.00
17	0.91	0.99
18	0.87	0.99
19	0.95	0.99
20	0.91	0.99
21	0.80	0.99
22	0.89	0.99
23	0.92	0.99
24	0.90	0.99
25	0.89	0.99
26	0.92	0.99
27	0.86	0.99
28	0.90	0.99
29	0.85	0.99
30	0.89	0.99
31	0.88	0.99
32	0.93	0.99
33	0.70	0.99
34	0.83	0.99
35	0.93	0.99
36	0.93	0.99
37	0.88	0.99
38	0.93	1.00
39	0.87	0.99
40	0.84	0.99
41	0.84	0.99
42	0.90	0.99
43	0.87	0.99
44	0.91	0.99
45	0.90	1.00
46	0.93	0.99
47	0.86	0.99
48	0.90	0.99
49	0.83	0.99
50	0.92	0.99
51	0.87	1.00
52	0.79	0.99
53	0.85	0.99
54	0.80	0.99
55	0.87	0.99

S11 The aromatic region

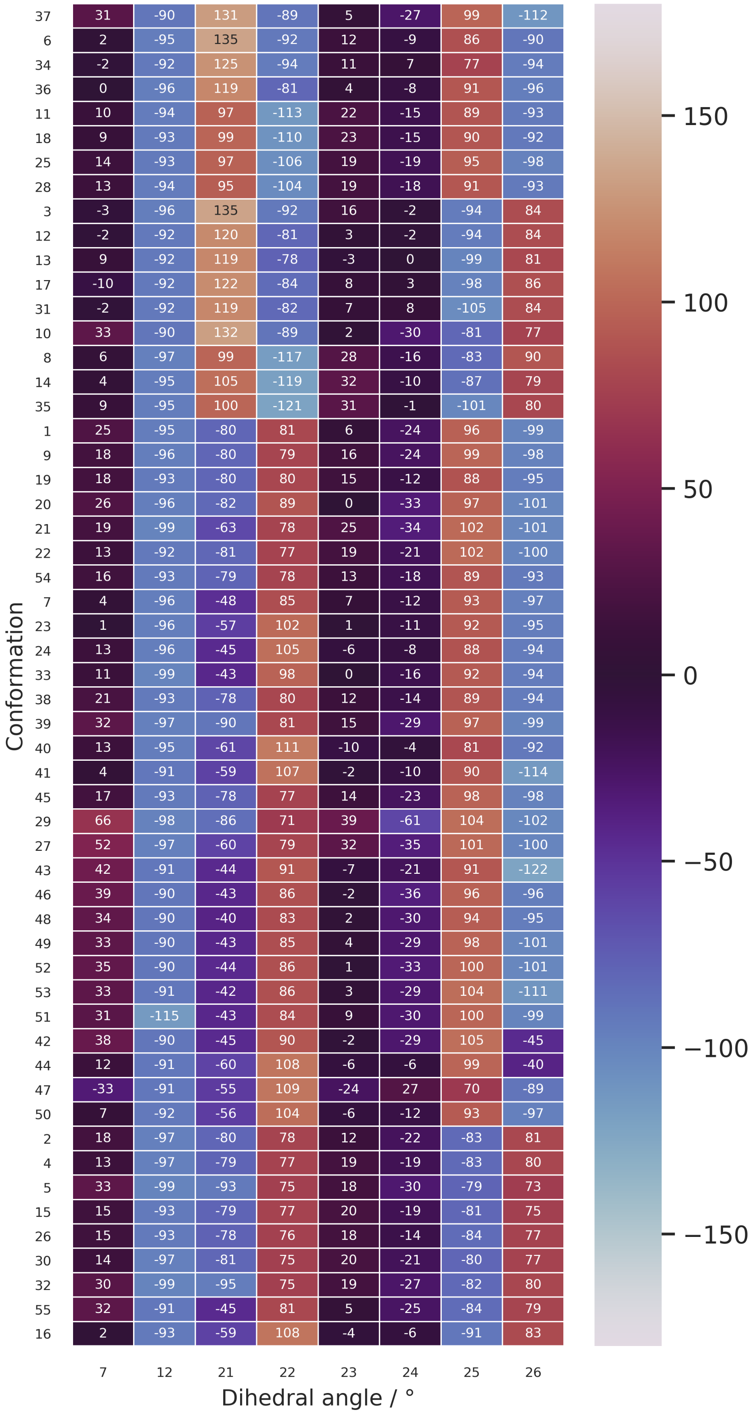

Fig. S20 A heatmap representation of the geometries behind the 55 conformations for a selection of dihedral angles (Fig. 2; main text). The conformations have been sorted to form conformational groups based on their presented dihedral angles.

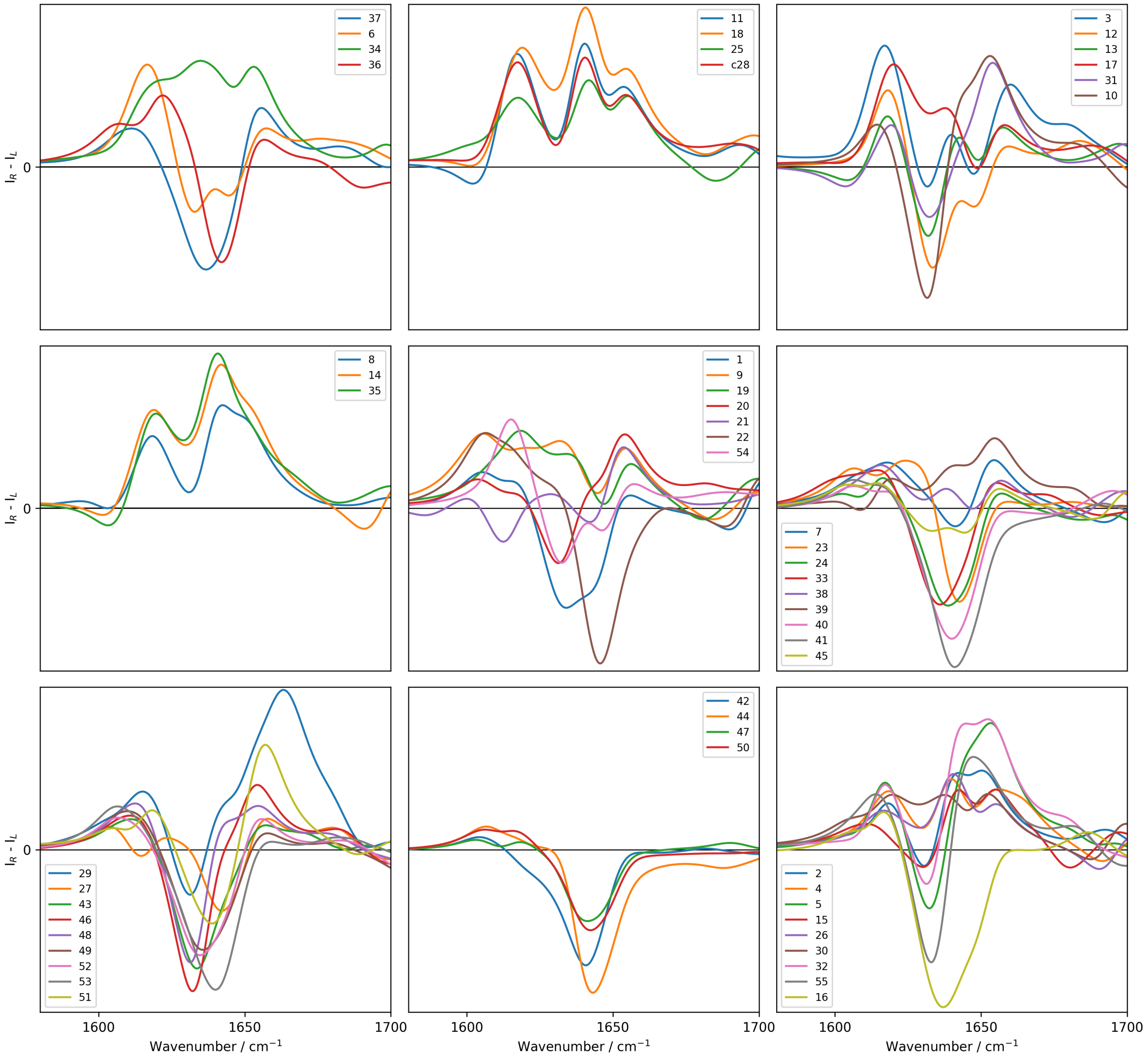

Fig. S21 The ROA spectra of all the 55 conformations in the aromatic region, grouped according to their conformations as sorted in Fig. S20. The conformation numbers are given in the legends.

References

[1] Y. Nitanai, T. Kikuchi, K. Kakoi, S. Hanamaki, I. Fujisawa and K. Aoki, *J. Mol. Biol.*, 2009, **385**, 1422–1432.

[2] W. G. Prowse, A. D. Kline, M. A. Skelton and R. J. Loncharich, *Biochemistry*, 1995, **34**, 9632–9644.

[3] S. G. Grdadolnik, P. Pristovšek and D. F. Mierke, *J. Med. Chem.*, 1998, **41**, 2090–2099.

[4] P. J. Loll, A. Derhovanessian, M. V. Shapovalov, J. Kaplan, L. Yang and P. H. Axelsen, *J. Mol. Biol*, 2009, **385**, 200 – 211.

[5] J. P. Mackay, U. Gerhard, D. A. Beauregard, R. A. Maplestone and D. H. Williams, *J. Am. Chem. Soc.*, 1994, **116**, 4573–4580.

[6] H. Goto and E. Osawa, *J. Am. Chem. Soc.*, 1989, **111**, 8950–8951.

[7] Kevin E. Gilbert, Pcmodel (version 10.0), Serena Software, Bloomington, IN, 2013.

[8] S. Grimme, C. Bannwarth, S. Dohm, A. Hansen, J. Pisarek, P. Pracht, J. Seibert and F. Neese, *Angew. Chem., Int. Ed.*, 2017, **56**, 14763–14769.

[9] T. A. Halgren, *J. Comput. Chem.*, 1996, **17**, 490–519.

[10] T. A. Halgren, *J. Comput. Chem.*, 1996, **17**, 520–552.

[11] T. A. Halgren, *J. Comput. Chem.*, 1996, **17**, 553–586.

[12] T. A. Halgren and R. B. Nachbar, *J. Comput. Chem.*, 1996, **17**, 587–615.

[13] T. A. Halgren, *J. Comput. Chem.*, 1996, **17**, 616–641.

[14] M. J. Frisch, G. W. Trucks, H. B. Schlegel, G. E. Scuseria, M. A. Robb, J. R. Cheeseman, G. Scalmani, V. Barone, G. A. Petersson, H. Nakatsuji, X. Li, M. Caricato, A. V. Marenich, J. Bloino, B. G. Janesko, R. Gomperts, B. Mennucci, H. P. Hratchian, J. V. Ortiz, A. F. Izmaylov, J. L. Sonnenberg, D. Williams-Young, F. Ding, F. Lipparini, F. Egidi, J. Goings, B. Peng, A. Petrone, T. Henderson, D. Ranasinghe, V. G. Zakrzewski, J. Gao, N. Rega, G. Zheng, W. Liang, M. Hada, M. Ehara, K. Toyota, R. Fukuda, J. Hasegawa, M. Ishida, T. Nakajima, Y. Honda, O. Kitao, H. Nakai, T. Vreven, K. Throssell, J. A. Montgomery, Jr., J. E. Peralta, F. Ogliaro, M. J. Bearpark, J. J. Heyd, E. N. Brothers, K. N. Kudin, V. N. Staroverov, T. A. Keith, R. Kobayashi, J. Normand, K. Raghavachari, A. P. Rendell, J. C. Burant, S. S. Iyengar, J. Tomasi, M. Cossi, J. M. Millam, M. Klene, C. Adamo, R. Cammi, J. W. Ochterski, R. L. Martin, K. Morokuma, O. Farkas, J. B. Foresman and D. J. Fox, *Gaussian˜16 Revision A.03*, 2016, Gaussian Inc. Wallingford CT.

[15] C. Mensch, L. D. Barron and C. Johannessen, *Phys. Chem. Chem. Phys.*, 2016, **18**, 31757–31768.

[16] J. R. Cheeseman and M. J. Frisch, *J. Chem. Theory Comput.*, 2011, **7**, 3323–3334.

[17] A. A. Osipov and L. M. Osipova, *J. Phys. Chem. Solids*, 2013, **74**, 971 – 978.

[18] L. D. Barron, F. Zhu, G. Hecht, G. E. Tranter and N. W. Isaacs, *J. Mol. Struc.*, 2007, **834-836**, 7–16.

[19] H. F. Boelens, R. J. Dijkstra, P. H. Eilers, F. Fitzpatrick and J. A. Westerhuis, *J. Chromatogr. A*, 2004, **1057**, 21–30.

[20] C. L. Covington and P. L. Polavarapu, *Chirality*, 2017, **29**, 178–192.

[21] M. A. Koenis, E. H. Tiekink, D. M. van Raamsdonk, N. U. Joosten, S. A. Gooijer, V. P. Nicu, L. Visscher and W. J. Buma, *Anal. Chim. Acta*, 2019, **1090**, 100 – 105.
